# Genome-wide assessment of the population structure and genetic diversity of four Portuguese native sheep breeds

**DOI:** 10.3389/fgene.2023.1109490

**Published:** 2023-01-13

**Authors:** Daniel Gaspar, Ana Usié, Célia Leão, Sílvia Guimarães, Ana Elisabete Pires, Claudino Matos, António Marcos Ramos, Catarina Ginja

**Affiliations:** ^1^ Centro de Biotecnologia Agrícola e Agro-Alimentar do Alentejo (CEBAL), Instituto Politécnico de Beja (IPBeja), Beja, Portugal; ^2^ BIOPOIS/CIBIO-InBIO, Centro de Investigação em Biodiversidade e Recursos Genéticos, Universidade do Porto, Vairão, Portugal; ^3^ MED—Mediterranean Institute for Agriculture, Environment and Development, Évora, Portugal; ^4^ Faculdade de Medicina Veterinária, Universidade Lusófona, Lisboa, Portugal; ^5^ ACOS—Agricultores do Sul, Beja, Portugal

**Keywords:** *Ovis aries*, high-throughput sequencing, population structure, genomic diversity, single nucleotide polymorphism, native breeds

## Abstract

As the effects of global warming become increasingly complex and difficult to manage, the conservation and sustainable use of locally adapted sheep breeds are gaining ground. Portuguese native sheep breeds are important reservoirs of genetic diversity, highly adapted to harsh environments and reared in low input production systems. Genomic data that would describe the breeds in detail and accelerate the selection of more resilient animals to be able to cope with climatic challenges are still lacking. Here, we sequenced the genomes of 37 animals from four Portuguese native sheep breeds (Campaniça, Bordaleira Serra da Estrela, Merino Branco and Merino Preto) and 19 crossbred sheep to make inferences on their genomic diversity and population structure. Mean genomic diversities were very similar across these breeds (.30 ≤ H_o_ ≤ .34; .30 ≤ H_e_ ≤ .35; 1.7 × 10^–3^ ≤ π ≤ 3.1 × 10^–3^) and the levels of inbreeding were negligible (.005 ≤ F_IS_ ≤ .038). The Principal Components, Bayesian clustering and Treemix analyses split the Portuguese breeds in two main groups which are consistent with historical records: one comprising Campaniça and Serra da Estrela together with other European and transboundary dairy breeds; and another of the well-differentiated multi-purpose Merino and Merino-related breeds. Runs of homozygosity analyses yielded 1,690 ROH segments covering an average of 2.27 Gb across the genome in all individuals. The overall genome covered by ROH segments varied from 27,75 Mb in Serra da Estrela to 61,29 Mb in Campaniça. The phylogenetic analysis of sheep mitogenomes grouped the Portuguese native breeds within sub-haplogroup B1a along with two animals of the Akkaraman breed from Turkey. This result provides additional support to a direct influence of Southwest Asian sheep in local breeds from the Iberian Peninsula. Our study is a first step pertaining to the genomic characterization of Portuguese sheep breeds and the results emphasize the potential of genomic data as a valid tool to guide conservation efforts in locally adapted sheep breeds. In addition, the genomic data we generated can be used to identify markers for breed assignment and traceability of certified breed-products.

## 1 Introduction

Since their domestication in the Fertile Crescent, approximately 10,500 years BP, sheep (*Ovis aries*) quickly became a valuable resource for the production of meat, milk, wool and leather products (Liu et al., 2016; Alberto et al., 2018). Nowadays, due to its physiological, morphological and behavioral characteristics, this species is well adapted to a wide range of climates and low-input agricultural environments. Local sheep are important domestic animal genetic resources for their biodiversity, role in landscape conservation and relevant contribution to the socio-economies of undeveloped and developing regions (Hassan et al., 1998; Yune and Abdela, 2017; Berihulay et al., 2019). The implementation of breeding strategies focused on environmental tolerance and specific traits of commercial interest, along with high mobility following transhumance routes, contributed to the high levels of biodiversity observed across the broad spectrum of sheep breeds worldwide (Kijas et al., 2012).

**FIGURE 1:**
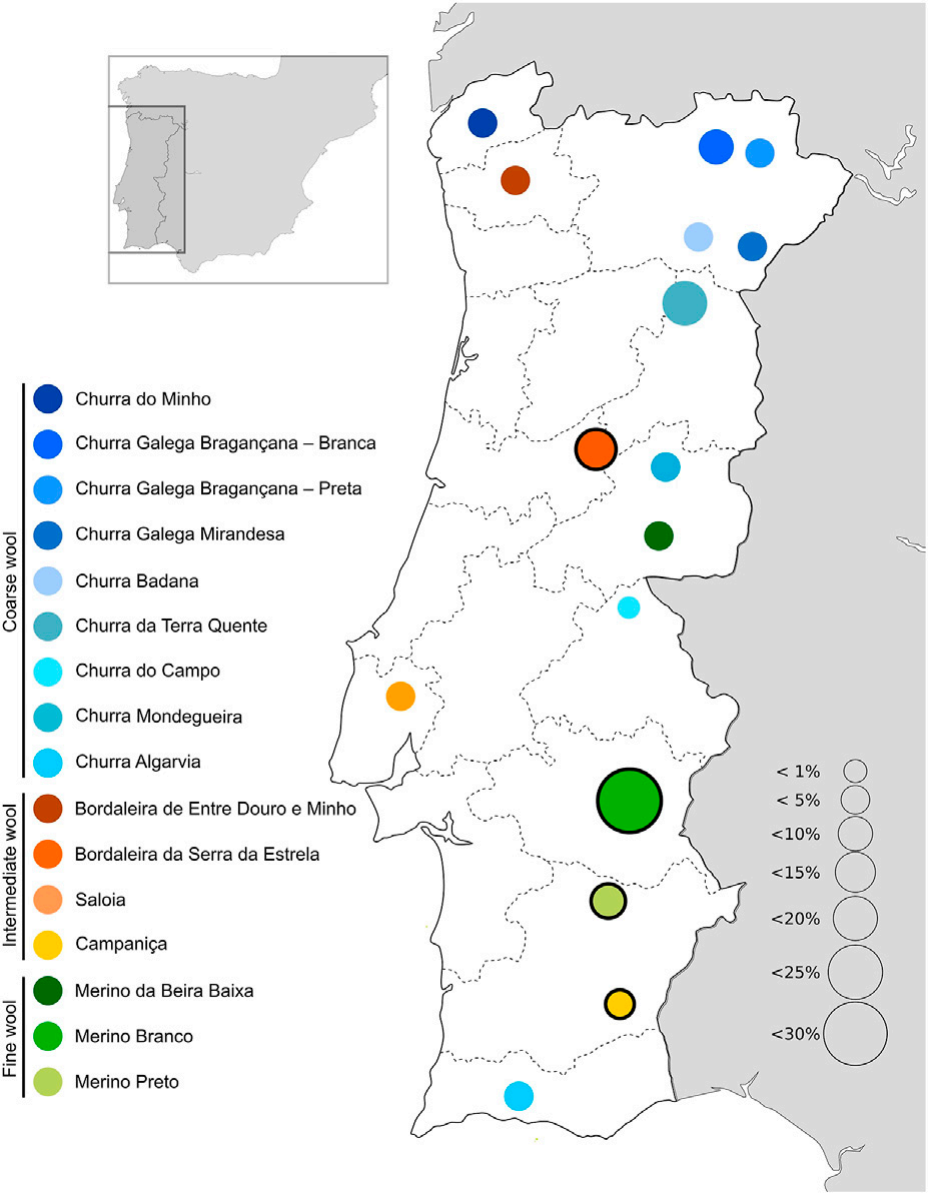
Geographical distribution and relative abundance of Portuguese native sheep breeds. The breeds sampled in this study are highlighted by a black circle.

In the Iberian Peninsula, sheep are common livestock reared across the territory mainly in agrosilvopastoral systems, contributing to the environmental sustainability and heritage value in rural communities (Dinis and Simões, 2021). Selection resulted in several breeds specialized for either meat, milk or wool production or reared as dual/triple purpose animals in distinct regions. In Portugal, there are 16 native sheep breeds registered in their specific herdbook (https://www.dgav.pt/animais/conteudo/recursos-geneticos-animais/racas-autoctones/ovinos/) (Figure 1). These breeds are divided in three major groups according to their fleece characteristics, i.e., Merino (fine wool), Bordaleiro (intermediate wool), and Churra (coarse wool) (Santos-Silva et al., 2008). Among the Portuguese sheep breeds, Bordaleira Serra da Estrela (SE), Merino Branco (MB), Merino Preto (MP), and Campaniça (CAM) are some of the most abundant raised under extensive conditions (Tiberio and Diniz, 2014) (Supplementary File S1). SE is the most important Portuguese dairy breed, inhabiting the Serra da Estrela Mountain region, one of the most inhospitable areas in the country. Its milk yield can exceed .78 L per day in a lactation period of up to 248 days. The Serra da Estrela cheese is a typical high-value product deriving from this breed, which has been granted a protected designation of origin (Carolino et al., 2003). However, the commercial value of this breed is not restricted to milk products. For many years, it was the wool provided by the SE herds that supplied the industry in this mountain region (Monteiro and Santos, 2021). The MB, MP and CAM breeds are mainly distributed in the south of Portugal, throughout the Alentejo region. They have shown an extraordinary ability to adapt to arid climates, thriving under harsh conditions and with poor food resources. Their intrinsic resilience and rusticity have been explored by breeders, creating opportunities to select animals that are better suited to cope with climate changes. These breeds produce high-quality meat, dairy and wool products (Matos, 2012a; Plowman et al., 2019). The population sizes of Portuguese native breeds have declined over the last years (Tiberio and Diniz, 2014), due to agricultural land abandonment and the consequent desertification, as well as replacement by more productive (but also more demanding) transboundary commercial breeds.

In the last decade, the enormous progress in high-throughput sequencing (HTS) technologies (Van Dijk et al., 2014) provides unprecedented opportunities for understanding the genomic basis for livestock phenotypic variability, including complex production traits (Zhang et al., 2011; Ghosh et al., 2018). The availability of HTS data allows to estimate genomic diversity and investigate the impact of demographic processes across the genome bringing in new perspectives for the conservation of local breeds (Fernández et al., 2016; Eusebi et al., 2020). Likewise, genome-wide single-nucleotide polymorphisms (SNPs) have been commonly used in commercial arrays to detect genetic variability in sheep breeds and describe their population structure (Kijas et al., 2009; Grasso et al., 2014). These markers are suitable for the detection of selection signatures across the genome based on, e.g., runs of homozygosity (ROH) (Peripolli et al., 2017), to detect genetic variants associated with traits of economic interest and to obtain valuable information to manage the extent of inbreeding in livestock breeds (Purfield et al., 2017). While molecular analyses have shown that native Iberian sheep hold great maternal haplotype diversity with three haplogroups (A, B, and C) represented (Pereira et al., 2006; Pedrosa et al., 2007; Chessa et al., 2009), genomic studies in these breeds are still lacking.

Genetic diversity is a key factor underlying the adaptive capacity and resilience of livestock populations under changing conditions (Hoffmann, 2013). The purpose of this study was to estimate the genomic diversity of four Portuguese native breeds and a population of crossbred sheep using a HTS approach. We also aimed to identify and characterize genome-wide patterns of ROH in these breeds. The HTS data was used to investigate the population structure of these Iberian breeds and their relationship with worldwide sheep. This is crucial to try to disclose their evolutionary histories and understand in which ways the changes in the population dynamics, e.g., bottlenecks and admixture, impacted their genomes and their differentiation. Our results have the potential of being used for an improved management of these local genetic resources, and the implementation of breeding strategies for the long-term conservation of Portuguese native sheep.

## 2 Materials and methods

### 2.1 Ethics statement

Animal handling and blood collection were performed during routine veterinary check-ups, following the official animal healthcare program guidelines and under the consent of breeders.

### 2.2 Biological samples and datasets

A total of 37 blood samples of animals representative of four Portuguese sheep breeds [Campaniça (*n* = 6), Serra da Estrela (*n* = 11), Merino Branco (*n* = 10) and Merino Preto (*n* = 10)] were collected at 15 farms throughout the country. Crossbred sheep (CB) sampled at other 10 farms distributed across the Alentejo region were also included in the analysis for comparison purposes [Crossbreds (*n* = 19)]. The animals were randomly selected from each herd. Ten milliliters of blood were collected from the jugular vein by vacuum puncture and stored at −20°C in collection tubes containing EDTA. Genomic DNA was extracted using the DNeasy Blood & Tissue Kit (Qiagen, Hilden, Germany) according to the manufacturer’s instructions. DNA concentration and quality were assessed with a Nanodrop spectrophotometer (Thermo Fisher Scientific^™^, Waltham, United States) and 500 ng of each sample were used for preparation of genomic libraries for resequencing (Protocols, 2022). Whole-genome HTS data were obtained through service acquisition (BGISEQ-500 sequencing platform, BGI, Shenzhen, China), which produced approximately 34.9 billion paired-end (2 × 100 bp) raw reads and an average depth of sequencing coverage of 21X. Details on locations, breed characteristics, sequencing statistics and accession numbers are shown in Supplementary Table S1.

We used two complementary strategies to investigate breed relationships and infer the population structure of Portuguese sheep breeds by integrating our HTS data with: 1) whole-genome data publicly available for other European, Asian, African, Australian and transboundary commercial breeds; and 2) Illumina Ovine 50 K SNP genotype data obtained for other Iberian breeds within the Sheep HapMap Consortium (International Sheep Genomics Consortium). The population structure analysis of worldwide sheep included a total of 48 animals representative of 18 breeds and the Asiatic mouflon (*Ovis orientalis*) which was used as an outgroup. Whole-genome HTS data were retrieved from the NCBI database (Bioproject ID: PRJNA624020; PRJNA160933; and PRJNA160933). Details on breeds, locations and accession numbers are shown in Supplementary Table S2. The SNP genotyping data consisted of 182 animals representing 9 Spanish breeds (Ciani et al., 2020) (see Supplementary Table S3 for details on breeds, sample sizes and locations). Furthermore, 48 mitogenomes retrieved from NCBI [Genbank accession n. NC_001941.1 (Hiendleder et al., 1998); PopSets 298110621 (Meadows et al., 2011), 583828744 (Lv et al., 2015) 158187235 (Burgstaller et al., 2007) and 528748432 (Lancioni et al., 2013)] were combined with mitochondrial consensus sequences from our shotgun data for a comprehensive phylogenetic analysis (see Supplementary Table S4).

### 2.3 Sequencing data pre-processing, mapping and SNP calling

The quality of paired-end raw reads was checked with the FastQC v.0.11.5 software (Andrews, 2010) and filtering was done with Trimmomatic v.0.38 (Bolger et al., 2014). Adapter sequences and low-quality bases, with less than an average quality threshold of 20 over a sliding window of 10 bp, were trimmed from the end of each read. Following, reads shorter than 80 bp were removed, resulting in ∼32.5 billion high-quality reads for downstream analyses. Mapping to the sheep reference genome Oar_rambouillet_v1.0 (Bioproject ID: PRJNA414087) was performed using BWA MEM v.0.7.15-r1140 (Li and Durbin, 2009) with default settings. The alignments were indexed and sorted with SAMtools v.1.4.1 (Li et al., 2009). Non-specific matches were excluded from the analysis, considering only unique mapped reads (91.8%) for SNP calling performed with Freebayes v.1.2.0 (Garrison and Marth, 2012). A total of 115,137,724 raw SNPs uniformly distributed across all chromosomes (*R* ^2^ = .966) were filtered based on quality (minQ > 30), SNP coverage per genotype (minDP ≥ 7) and genotype quality (minGQ > 20) using VCFtools v.0.1.17 (Danecek et al., 2011). After filtering, a set of 31,320,380 high-quality autosomal SNPs was used for downstream analyses. SNPs were then annotated using ANNOVAR (downloaded 2019-10-24) (Wang et al., 2010) and categorized according to the functional effects and distribution across genomic regions that included X (65.2%), Y (33.4%) and Z (.7%) located within intergenic, intronic and exonic regions, respectively. The SNPs found in coding regions, included 120,172 (57.2%) and 80,882 (38.5%) associated with synonymous and non-synonymous effects, respectively (Supplementary Table S5).

### 2.4 Genomic diversity and differentiation

Estimates of genomic diversity and the levels of differentiation among Portuguese sheep breeds were determined from HTS data with VCFtools v.0.1.17 (Danecek et al., 2011), in particular: nucleotide diversity (π); observed and expected heterozygosities (H_O_ and H_E_, respectively); genomic inbreeding coefficient (F_IS_) and fixation index (F_ST_). First, autosomal SNPs were filtered based on MAF (--maf .05), calling rate (--max-missing .1) and Hardy-Weinberg equilibrium (--hwe .001). SNPs that did not pass these quality criteria were excluded from the analysis. The nucleotide diversity was estimated as the average number of nucleotide differences per site within 10 Kb windows (--window-pi 10,000) across the genome. The expected and observed heterozygosities, as well as the inbreeding coefficients, were estimated for each population using the functions (--hardy) and (--het), respectively. For pairwise breed comparisons, F_ST_ values were calculated following Weir and Cockerham’s (Weir and Cockerham, 1984), with a sliding window of 10 Kb.

### 2.5 Detection and distribution of runs of homozygosity

A genome-wide detection of runs of homozygosity (ROH) was carried out in Portuguese native sheep in a sliding-window approach using PLINK software v.1.90b5.2 (Purcell et al., 2007). Briefly, ROH were defined as homozygous segments longer than 1 Mb and containing at least 50 autosomal SNPs with an average density of more than one SNP per 100 Kb. Furthermore, a segment was considered a ROH, if there was up to one heterozygous loci, no more than five missing genotypes and a maximum gap between consecutive SNPs of 250 Kb. The detected ROHs were categorized based on their length, and consensus ROH segments were estimated for each breed. Finally, the ROH-based inbreeding coefficient (F_ROH_) was calculated either for each chromosome or genome-wide in each population as the ratio of the total length of ROH for each individual and the total length of the autosomal chromosomes. The R v.4.0.5 (Team, 2020) package detectRUNS v.0.9.6 (https://CRAN.R-project.org/package=detectRUNS) was used to obtain summary statistics and visualize the results.

### 2.6 Population structure of Iberian and worldwide sheep breeds

PLINK v.1.90b5.2 was used to carry out principal component analysis (PCA) and integrate the autosomal HTS data obtained for the Portuguese sheep with: 1) whole-genomes of 45 worldwide sheep and three Asian mouflon (*Ovis orientalis*) (Supplementary Table S2); and 2) SNP genotyping data available for 182 native sheep from Spain (Supplementary Table S3). To detect first-degree relationships between individuals, KING kinship coefficients (Manichaikul et al., 2010) were estimated using a cutoff of .177. The combined data sets were pruned according to the following: remove SNPs with a minor allele frequency (MAF) lower than 5%; exclude samples and markers with more than 10% missing data; account for Hardy-Weinberg equilibrium (-hwe .001) and linkage disequilibrium (--indep-pairwise 50 10 2). A total of 987,574 and 19,651 autosomal SNPs were retained for downstream analyses, respectively. Additionally, population structure was also assessed using the model-based clustering approach implemented in ADMIXTURE v.1.3.0 (Alexander and Lange, 2011). Individual ancestry proportions were calculated for *K* values ranging from 2 to 21 using the default settings. For each K value, five replicate runs with different random seeds were done. The CLUMPAK software (Kopelman et al., 2015) was used to infer the most likely K based on Evanno et al. (2005) and considering K values from 2 to 14. A graphical representation of these results was obtained using the Tidyverse collection of the R packages (Wickham et al., 2019).

### 2.7 Phylogenetic analyses of autosomal and mitogenome data

The Maximum Likelihood (ML) phylogeny of sheep mitogenomes was inferred under the TN93 + R evolutionary model selected using the Akaike Information Criterion (AIC) (Posada and Buckley, 2004) in the PhyML software v.3.0 (Guindon et al., 2010) online platform, starting tree with BioNJ and branch support calculated from 100 bootstrap inferences. Briefly, after clean reads mapping to the reference sheep mitogenome, the consensus sequences were retrieved with ANGSD v.0.935 (-doFasta 3 -minQ 20 -minMapQ 30 -MinDepth 7 -doDepth 1) (Korneliussen et al., 2014) in FASTA format. Representatives of each maternal haplogroup available from public repositories were included in this analysis (Supplementary Table S4). A total of 104 mitogenomes were aligned using MUSCLE v.3 (Edgar, 2004). Finally, the phylogenetic tree was visualized and edited in FigTree v.1.3.1 (http://tree.bio.ed.ac.uk/software/figtree/).

The Treemix software v.1.13 (Pickrell and Pritchard, 2012) was used to investigate genetic relationships (splitting and mixing) between Iberian sheep breeds using allele frequencies for 11,239 SNP positions included in the Illumina Ovine 50 K SNP array that can be unambiguously assigned to autosomal positions in the sheep reference genome Oar_rambouillet_v1.0 (Bioproject ID: PRJNA414087) following (Nicolazzi et al., 2015). Treemix was run using the default settings with a block size of 100 SNPs, 500 bootstrap replicates and Asian Mouflon as outgroup. The optimal number of migration events (*m* = 1–10) to add to the tree were determined using the OptM package (Fitak, 2021) in R v.4.0.5 (Team, 2020) with 10 independent replicates at each value of m. Phylogenetic networks were visualized using the Treemix R script “plotting_funcs.”

## 3 Results

### 3.1 Genomic diversity

**FIGURE 2:**
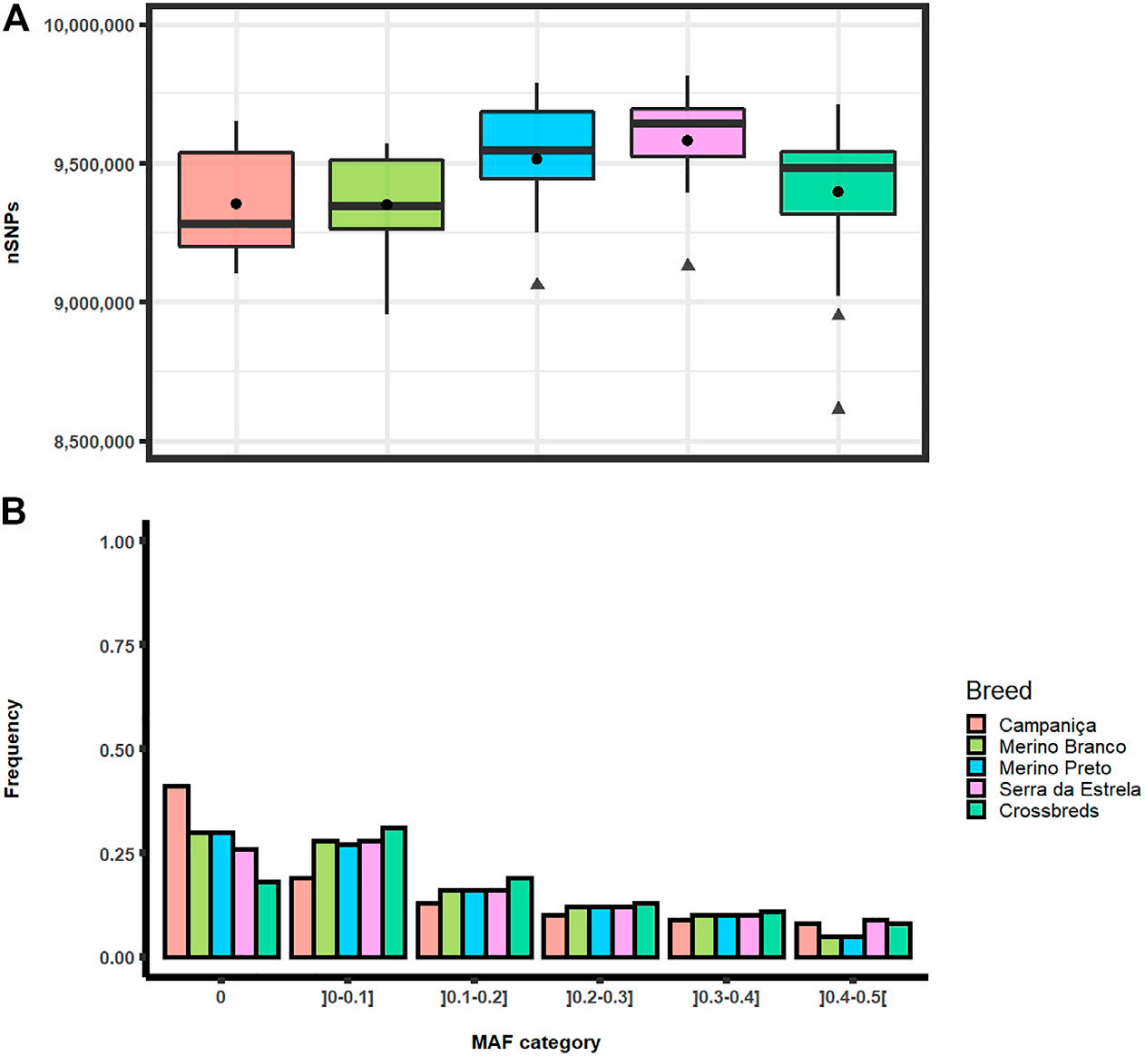
Distribution of single nucleotide polymorphisms in Portuguese sheep. **(A)** Boxplot graph of the total number of SNPs per individual observed in each population. Mean values are represented by black circles and outliers by triangles; **(B)** Frequency of minor allele frequency (MAF) by category in each breed.

Analyses of genomic diversity based on HTS data were performed for 56 sheep of four Portuguese native breeds (Campaniça, Merino Branco, Merino Preto and Bordaleira Serra da Estrela) and the crossbred sheep population. On average, the total number of SNPs per individual ranged from 9,348,138 SNPs in Merino Branco to 9,581,638 SNPs in Serra da Estrela. Based on the Kruskal-Wallis statistical inference analysis for the *p*-value cutoff of .01 the average number of SNPs per individual did not differ significantly between breeds (Figure 2A).

The distributions of SNPs across MAF categories in each population are summarized in Figure 2B and are useful to evaluate the gene pool richness and genomic variability. The proportion of fixed SNPs (MAF = 0) displayed considerable differences among breeds, with CAM showing a markedly higher percentage (41.5%) than the other breeds, whereas the percentage of highly polymorphic SNPs (.4 < MAF <.5) was more uniform across breeds (overall average of 6.7%) ranging from 4.9% in MB and MP to 8.6% in SE. The overall levels of genomic variability were very similar across these sheep breeds (Table 1). On average, autosomal nucleotide diversity ranged from π = 1.956 × 10^–3^ (SE) to π = 2.032 × 10^–3^ (MP). The comparisons between breeds of expected (He) and observed (Ho) heterozygosities were not significantly different (Kruskal-Wallis, *p* < .01). On average, He varied between .3 (MB and MP) and .35 (CAM) with an overall mean of .32, and Ho ranged from .30 (MB and MP) to .34 (CAM and SE) with an overall mean of .31. The genomic inbreeding coefficient estimated from SNP data was relatively low in all breeds (MB: .012, MP: .005 and SE: .022), with the highest value observed in CAM (.038). Weir and Cockerham’s mean pairwise F_ST_ was used as a measure of breed differentiation across breeds (Table 2). The pairwise-breed F_ST_ values ranged from .005 (MB and CB) to .037 (CAM and MB), showing a close genetic relationship between Portuguese native sheep. CAM and the dairy breed SE had the highest mean F_ST_ across pairwise comparisons, consistently with their relative geographic isolation, and in the case of the latter also selection for milk production.

**TABLE 1:** Genomic diversity of Portuguese sheep. Breed names and acronyms, sample sizes, observed (Ho) and expected (He) heterozygosity, nucleotide diversity (π) and the inbreeding coefficient (F_IS_) are shown.

Breed name	Acronym	Sample size	Genomic diversity indexes			
			H_o_	H_e_	π	F_IS_
Campaniça	CAM	6	.34	.35	2.016 × 10^–3^	.038
Merino Branco	MB	10	.30	.30	2.006 × 10^–3^	.012
Merino Preto	MP	10	.30	.30	2.032 × 10^–3^	.005
Bordaleira Serra da Estrela	SE	11	.34	.35	1.956 × 10^–3^	.022
Crossbreds	CB	19	.30	.31	1.981 × 10^–3^	.026

**TABLE 2:** Pairwise-breed estimates of genomic differentiation (F_ST_) among Portuguese sheep. Breed acronyms are shown in Table 1.

Breed	CAM	MB	MP	SE	CB
CAM	—				
MB	.037	—			
MP	.035	.021	—		
SE	.028	.029	.027	—	
CB	.035	.005	.020	.027	—

### 3.2 Runs of homozygosity and inbreeding

**FIGURE 3:**
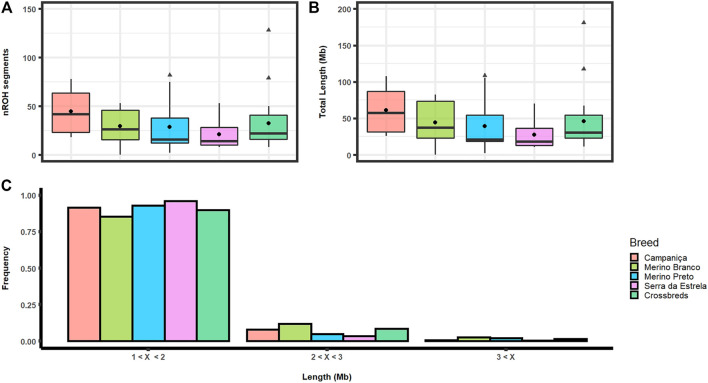
Runs of homozygosity (ROH) in Portuguese sheep. **(A)** Boxplot graph depicting the average number of ROH segments (nROH) per animal in each population. Mean values are represented by black circles and outliers by triangles; **(B)** Boxplot graph depicting average ROH length in each population. Mean values are represented by black circles and outliers by triangles; **(C)** Frequency distribution of the number of ROH by different length categories, i.e., short (1–2 Mb), medium (2–3 Mb) and large (over 3 Mb) for each population.

We identified a total of 1,690 ROH comprising an average of 2.27 Gb across the genome in Portuguese sheep. The number of ROH ranged from 11 on chromosome 24 to 213 on chromosome 3. The distribution of ROH segments was strongly correlated with the chromosome size (*R* ^2^ = .8239). CAM showed the highest average number of homozygous segments per animal (nROH = 44.5) comprising on average 61.29 Mb, whereas the lowest values were observed in the dairy breed SE (nROH = 20.9) comprising about 27.75 Mb per animal. The Merino breeds showed an intermediate number of ROH segments (MB: 29.5 and MP: 28.5) and mean lengths (MB: 44.39 Mb, and MP: 39.53 Mb) per animal (Figures 3A, B). The longest homozygous segment (∼4.9 Mb harbouring 27,839 SNPs) was identified on chromosome 6 in MB. The ROH segments were grouped in three categories by length: 1) short (1 Mb–2 Mb); 2) medium (2 Mb–3 Mb); and 3) Large (>3 Mb). Most of these ROH belong to the short category (∼91%), while the large category accounted for a small fraction (∼1.6%) (Figure 3C).

**FIGURE 4:**
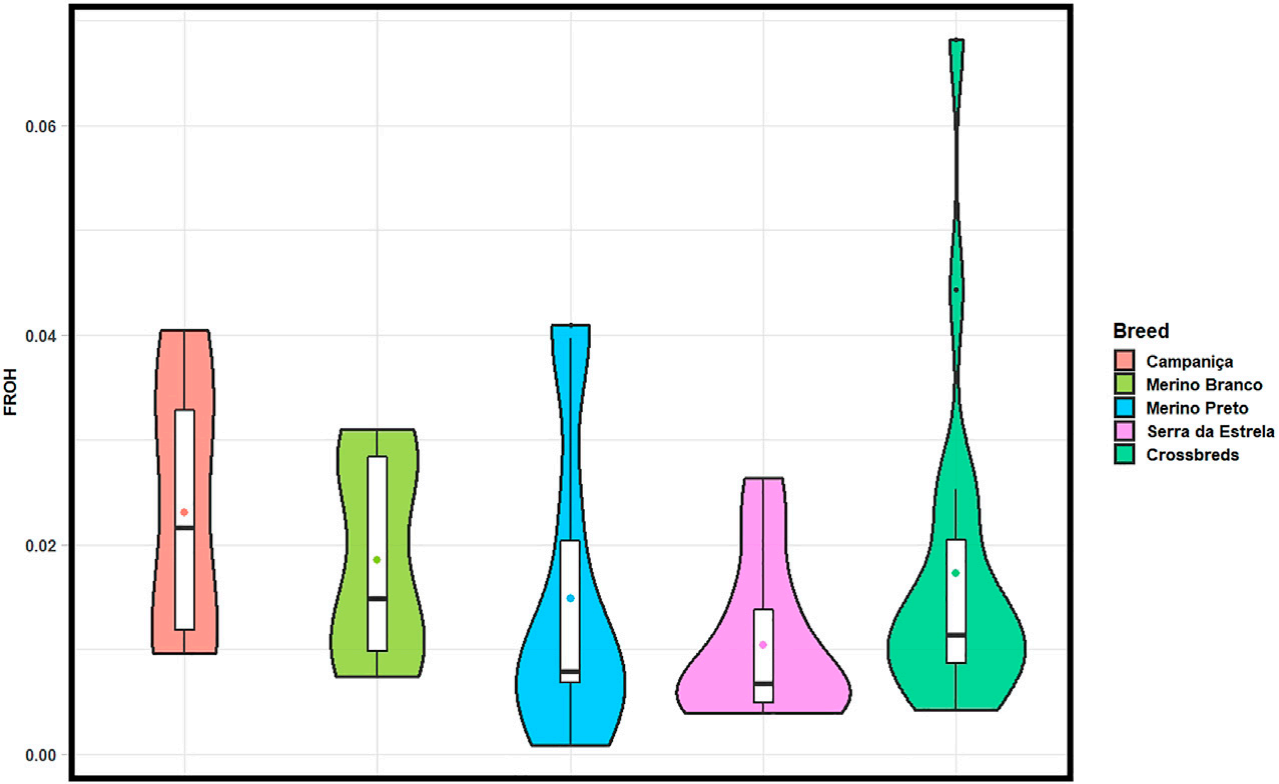
Violin plots showing ROH-based inbreeding coefficient (FROH) calculated in each population considering all ROH segments (> 1 Mb). Mean values are represented by coloured circles.

The ROH-based inbreeding coefficient (FROH) was estimated for each population (Figure 4). On average, CAM had the highest F_ROH_ (.023) and SE the lowest (.010). F_ROH_ estimates across chromosomes varied within and between breeds (Supplementary Figure S1), which suggests that it could be associated to regions under positive selection for specific production or adaptive traits.

### 3.3 Population structure in Iberian and worldwide sheep

**FIGURE 5:**
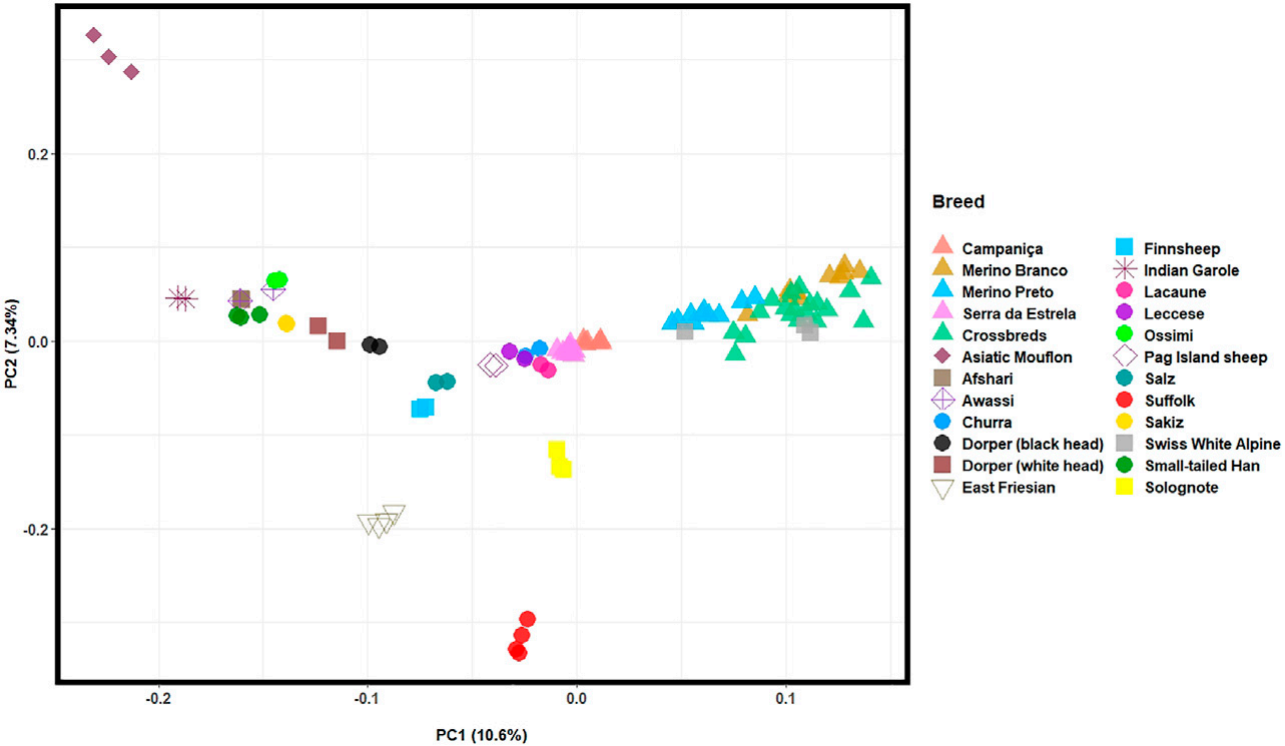
Principal component analysis (PCA) of Iberian and worldwide sheep. PC1 and PC2 account for 10.3% and 7.27% of the total genomic variation, respectively. The Portuguese sheep are represented by coloured triangles, as follows: Campaniça in salmon; Merino Branco in golden brown; Merino Preto in blue; Serra da Estrela in pink; and Crossbreds in green. See Supplementary Table S2 for details on each individual included in the analysis.

The PCA based on HTS data was conducted to assess the population structure of Portuguese native and worldwide sheep breeds and infer the proportion of the total genomic variation explained by each PC. The first two PCs, which account for the highest variation of the data set, depicted: an east-to-west cline of sheep breeds (PC1, explains 10.3% of the total genetic variation); and the differentiation between the Asiatic Mouflon, domestic sheep and the inbred Suffolk and East-Friesian breeds (PC2, 7.3%) (Figure 5). The Portuguese populations clustered into two groups, as follows: 1) the well-differentiated Merino breeds grouped together with the Merino-derived Swiss White Alpine breed indicating a close genetic relationship; 2) CAM and SE clustered in the central group along with other European and dairy sheep breeds.

**FIGURE 6:**
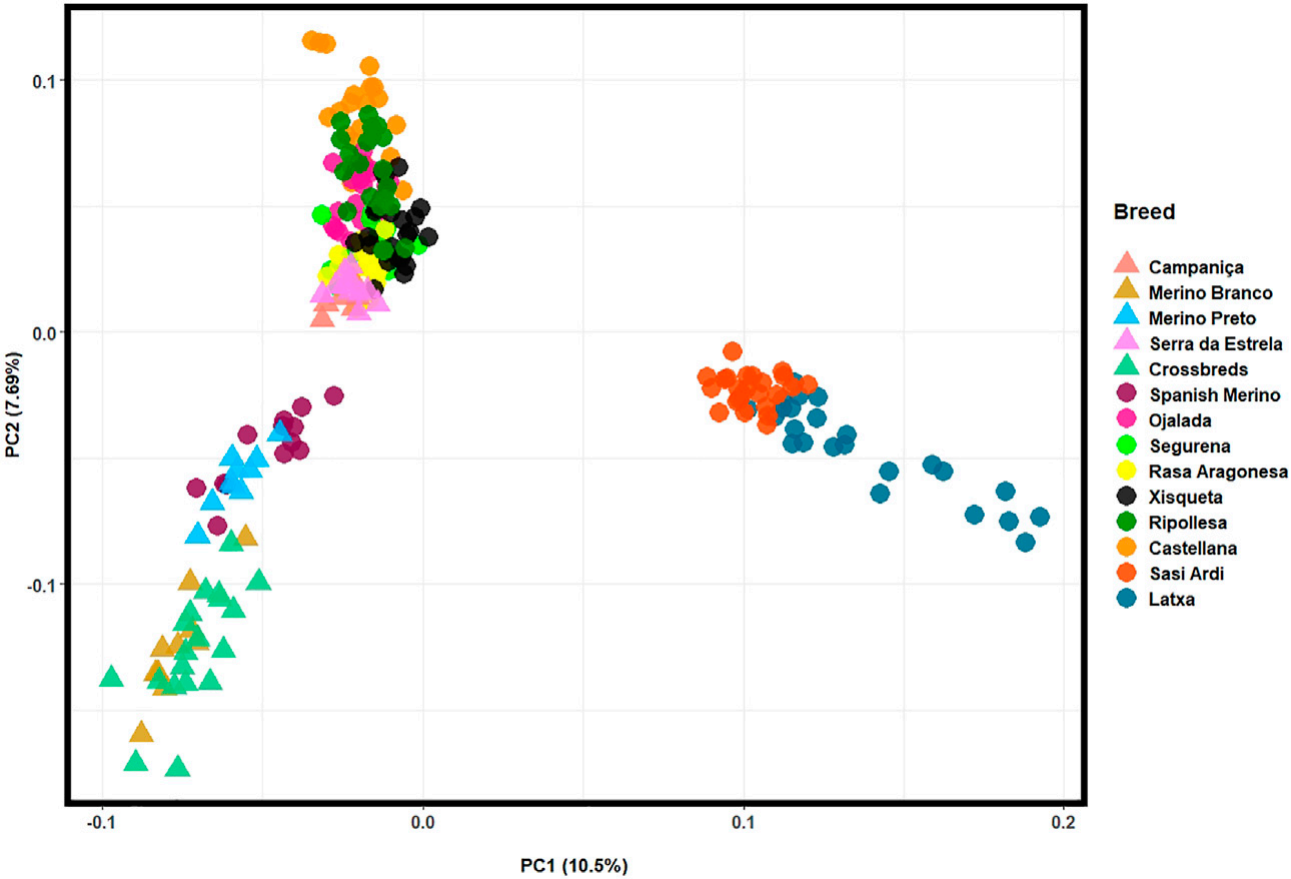
Principal component analysis (PCA) of Iberian sheep. PC1 and PC2 account for 10.5% and 7.69% of the total genomic variation, respectively. Portuguese sheep are represented by coloured triangles, as follows: Campaniça in salmon; Merino Branco in golden brown; Merino Preto in blue; Serra da Estrela in pink; and Crossbreds in green. See Supplementary Table S3 for details on each individual included in the analysis.

For a fine resolution, the population structure of Iberian breeds was also assessed in a PCA by merging our HTS data collected for Portuguese sheep with Illumina 50 K SNP genotyping data available for nine Spanish breeds (Figure 6). The first two components accounted for over 18% of the total genomic variation. The Portuguese Merino breeds and the crossbreds clustered together with the Spanish Merino, which confirms their close genetic relationship. In addition, CAM and SE were separated from the Merino cluster by PC2 and grouped with other intermediate-fine wool Spanish breeds. The Basque breeds of coarse wool type—Latxa and Sasi Ardi, formed an isolated cluster.

**FIGURE 7:**
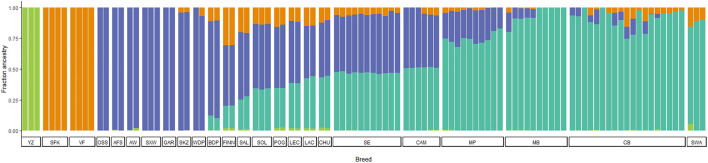
Model-based clustering analysis of Iberian and worldwide sheep. The proportions of the inferred ancestral clusters (K = 4) are depicted by the different colours with each individual represented by a bar and sorted by breed. CAM—Campaniça; MB—Merino Branco; MP—Merino Preto; SE—Serra da Estrela; and CB—Crossbreds. The results for K = 2 to K = 21 are shown in Supplementary File S2. See Supplementary Table S2 for details on each individual included in the analysis.

ADMIXTURE analysis allowed us to infer ancestry contributions underlying the gene pool of Portuguese native sheep. The results of the model-based clustering approach are shown in Figure 7 for the most likely K value (K = 4) (for the Delta K graph see Supplementary Figure S2). Additional results were obtained for values of K ranging from K = 2 to K = 21 (Supplementary File S2). When considering two ancestral populations (K = 2), CAM and SE shared a greater proportion of the mouflon component along with other European and dairy breeds, than Merino and the SWA breeds which had little contributions. For K = 3, the mouflon formed an independent cluster, with the Asian and Middle Eastern sheep showing some proportion of mouflon ancestry. For K = 4, the Merino sheep and the SWA clustered together and were more homogeneous than their Iberian counterparts CAM, SE and CHU (a coarse wool breed) which showed an admixed ancestry. Crossbreds shared MB and SWA ancestry. Also, the Asian sheep formed their own group, while VF and SFK were clearly differentiated from all other breeds. As K-values increased, CAM and SE individuals split in two clusters, with three individuals from each of these breeds showing a more heterogeneous pattern of ancestry common to other European populations (CHU, LAC, LEC, POG, and FINN). For K > 9, MP forms a separate cluster from MB, SWA and the crossbreds. These patterns of ancestry were also observed when the crossbreds were removed from the analysis (results not shown).

### 3.4 Phylogenetic analyses of mitogenome and autosomal data

**FIGURE 8:**
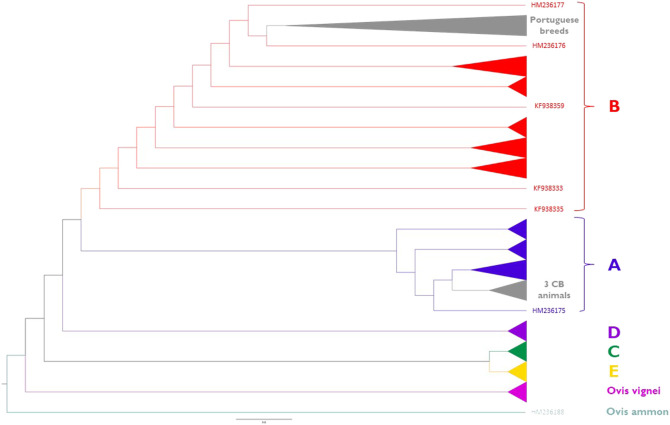
Maximum-Likelihood phylogeny of sheep mitogenomes. Portuguese native sheep (light grey) belong to haplogroup B1a and three crossbred animals clustered within haplogroup A1a. Reference sequences representing major haplogroups are colored as follows: **(A)** (dark blue); **(B)** (red); **(C)** (dark green); **(D)** (purple); **(E)** (yellow). Wild sheep mitogenomes were also included in the analysis: *Ovis musimon* (within haplogroup A); *Ovis vignei* (pink); *Ovis ammon* (petrol blue). See Supplementary Table S4 for details on each individual included in the analysis.

Phylogenetic relationships inferred from the sheep mitogenomes are depicted in Figure 8 (for details see Supplementary Figure S3). Portuguese native breeds belong to haplogroup B except for three crossbred animals that were assigned to haplogroup A. We did not observe a clear differentiation between the maternal lineages of the fine-wool (MB and MP) and intermediate-wool (CAM and SE) breeds. Portuguese native breeds formed a well-supported cluster within sub-haplogroup B1a along with two individuals of the Akkaraman breed from Turkey (Genbank acc. n. HM236176 and HM236177) and the reference mitogenome of a Merinolandschaf (Genbank acc. n. NC001941).

**FIGURE 9:**
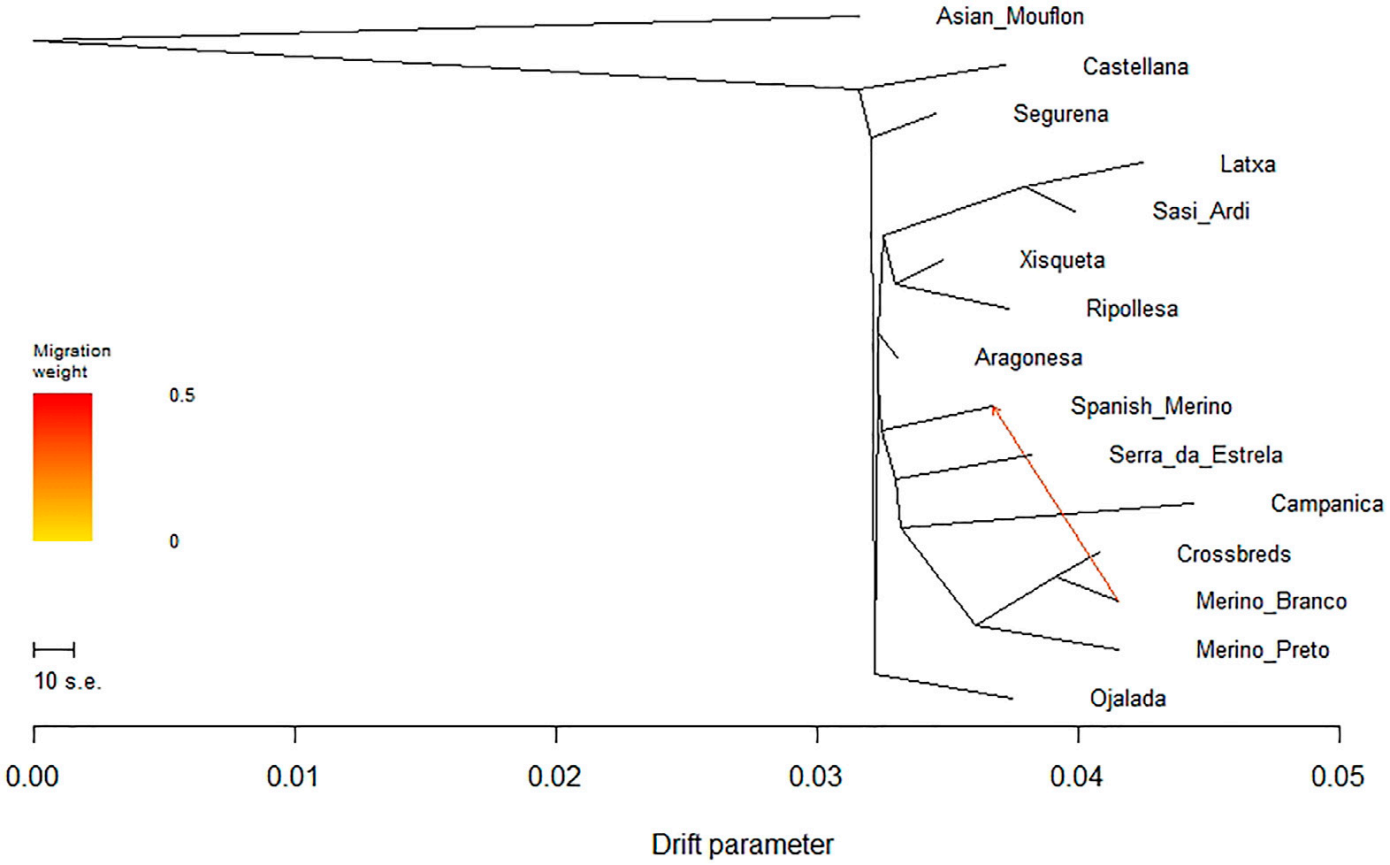
Phylogenetic network inferred by Treemix for Iberian sheep. One migration event (m = 1) among populations was allowed and is represented by an arrow indicating gene flow between Merino Branco and Spanish Merino.

To evaluate the phylogenetic relationships and historical genetic drift events among Iberian breeds, we built a maximum likelihood (ML) tree based on the population allele frequency covariance matrix and rooted in the Asian Mouflon using TreeMix (Supplementary Figure S4). When one migration event was assumed, all domestic sheep populations clustered into one primary branch, showing the presence of gene flow between Merino Branco and Spanish Merino (Figure 9). Overall, the topology of the ML tree was consistent with the results revealed by the PCA analysis.

## 4 Discussion

We conducted the first whole-genome sequencing analysis of Portuguese native sheep in which four breeds and a population of crossbreds were characterized in the context of other Iberian and worldwide sheep. Improving knowledge on the genomic diversity and population structure of local sheep is especially important to disclose signatures of adaptation and improvement, but also to implement appropriate management and conservation strategies. To date, few genetic studies have been carried out on Portuguese native sheep and were based on either mitochondrial data or a limited number of microsatellite markers (Pereira et al., 2006; Santos-Silva et al., 2008; Landi et al., 2019). We report genomic variation and ROH patterns in these breeds. Following, we used complementary population genetics and phylogenetic approaches to infer population structure, patterns of admixture and breed relationships.

Genetic variation among breeds is usually expressed in terms of allele frequencies. Our results revealed a moderate level of polymorphic SNPs (MAF >.01) with slight differences between breeds. However, a significantly high proportion of fixed SNPs (MAF = 0) was observed in CAM which can be due to a recent bottleneck from replacement by high-output transboundary breeds (Matos, 2012b), or to the fact that samples were collected in two herds. In addition, the proportion of polymorphic SNPs in Portuguese Merino breeds was lower (70% in Merino Branco and Merino Preto) than in the Merino populations analyzed by Grasso et al. (2014) (89,4%), but this might be because they used the OvineSNP50 BeadChip (Illumina) in a large number of animals. Genetic diversity is fundamental to strengthen the ability of populations to evolve to adapt to changes. Overall, our results revealed similar levels of genomic diversity across the studied breeds. The observed and expected heterozygosity values (from .30 to .34 and from .30 to .35, respectively) were slightly lower in Merino breeds. Non-etheless, they were consistent with those reported for other European breeds (.30 ≤ H_o_ ≤ .39; .31 ≤ H_e_ ≤ .38) (Luigi-Sierra et al., 2019). Nucleotide diversities were also comparable to those observed for a wide range of sheep breeds (varying from 1.7 × 10^–3^ to 3.1 × 10^–3^) (Lv et al., 2022).

In livestock populations, high inbreeding could result in an overall decrease of their performance, which may impact economically important traits (Leroy, 2014). For instance, there is evidence that high levels of inbreeding have detrimental effects in the growth of lambs (Černá et al., 2021). Inbreeding coefficients (F_IS_) estimated for the Portuguese native breeds were relatively low (ranging from .005 to .038). Even the highest F_IS_ value estimated for CAM is considerably lower than the observed in some European sheep breeds (between .04 and .42) (Kijas et al., 2012). As opposed to most commercial breeds, farmed under intensive conditions and subjected to intensive selection programs, Portuguese native breeds are reared by smallholder farmers in traditional agrosilvopastoral systems, where random mating predominates and admixture between flocks may occur. This might explain the low levels of inbreeding observed, as well as the somewhat low genetic differentiation between breeds (mean pairwise F_ST_ .03).

The patterns of ROH can help discriminate ancient bottlenecks (i.e., many short ROHs) from recent inbreeding and low genetic diversity (i.e., few long ROHs) (Curik et al., 2014). In our study, shorter ROH segments (1–2 Mb) were found far more frequently than longer ones (>2 Mb). The frequency of these short ROH was equal or greater than 90% in all breeds, except in MB. Our estimates are within the reported range for other local breeds (Al-Mamun et al., 2015; Deniskova et al., 2021; Liu et al., 2021), including Spanish sheep (Luigi-Sierra et al., 2019), suggesting recent autozygosity events were not frequent in the Portuguese breeds analyzed. In the absence of pedigree records, ROH has been widely used to estimate inbreeding (F_ROH_) with a large number of SNPs (Kardos et al., 2015). The F_ROH_, defined as the proportion of the autosomal genome covered by ROHs, was generally low in all breeds (.010 ≤ F_ROH_ ≤ .023). The F_ROH_ values obtained for Portuguese sheep were similar to those reported for the Spanish breeds Castellana, Ojalada, Ripollesa, Segurena and Xisqueta (.008 ≤ F_ROH_ ≤ .025) (Luigi-Sierra et al., 2019). Overall, F_ROH_ values agreed with the relative abundance and length of ROH, i.e., CAM had the highest number of ROH segments, the largest proportion of the genome covered by ROH and consequently the highest F_ROH_ value. The lowest F_ROH_ was observed in SE, which also displayed the lowest ROH counts and lowest length of the genome covered by ROH per individual. It is not surprising that SE animals sampled in nine herds are less related to each other than those of other breeds that derive from only 3 to 4 farms.

Population structure and breed relationships were investigated considering worldwide and Iberian sheep breeds by integrating the genomes we generated with publicly available whole-genome and SNP array data, respectively. Congruent results were obtained from complementary PCA, Admixture and phylogenetic analyses. In the PCA, Portuguese sheep split in two clusters according to breed histories, i.e., Merino populations were genetically close whereas CAM and SE belong to a distinct group of breeds. The Swiss White Alpine is a Merino-derived European breed and in agreement with previous analysis was also included in this group (Ciani et al., 2020). When only Iberian sheep were considered, the Portuguese Merino breeds grouped together in the PCA, along with their Spanish counterparts. These breeds share a common genetic background that could result to some extent from their geographic proximity (Landi et al., 2019). The CAM and SE breeds belong to a more heterogeneous group that included other Iberian breeds such as Churra from Spain, as well as other European sheep raised for milk (e.g., Lacaune originally from France), meat (e.g., Pag Island sheep from Croatia upgraded with Merino), or as dual-purpose animals (e.g., Leccese from Italy). The admixed background of some of these breeds has been interpreted as the consequence of ancient gene flow along the Mediterranean (Lv et al., 2015; Ciani et al., 2020). In the Iberian context, sheep breeds are typically classified according to the characteristics of their fleece (Pedrosa et al., 2007). Interestingly, the PCA clustering clearly depicted fine wool Merino breeds separated from intermediate and coarse-wool sheep from Portugal and Spain, including CAM and SE. Coarse wool sheep from the Basque region were highly differentiated from all other breeds probably due to their geographical isolation.

The ADMIXTURE analysis allowed us to investigate in more detail the ancestry components underlying the genetic structure of Portuguese native breeds. For low values of K, general patterns of ancestry could be inferred, such as the clear differentiation of the Asiatic mouflon and of more commercial breeds (e.g., Suffolk and East Friesian), as well as the tight clustering of the Portuguese Merino populations. For K greater than six, some sub-structure within CAM, SE, MP, and MB starts to emerge, which could be due to an effect of the herds from where the samples originate. Overall, our results are consistent with low levels of breed differentiation and a complex genetic background observed in sheep from the Iberian Peninsula (Luigi-Sierra et al., 2019). The weak differentiation observed between MB and CB suggests that this crossbred population resulted mainly from the mating of local Merino type animals with high performance individuals from transboundary commercial breeds. This result was also observed for an admixture analysis of the Iberian dataset, which corroborated the PCA clusters shown in Figure 6, i.e., the close affinities between Portuguese Merino, Spanish Merino and the crossbreds (results not shown). In the first half of the 20th century and according to the MB Breeder’s Association (https://www.merina.pt), national authorities promoted the upgrading of local Merino sheep with Merino animals from Spain, as well as Rambouillet and Merino Precóce from France. The Rambouillet is itself derived from Spanish Merino flocks (http://afs.okstate.edu/breeds/sheep/rambouillet), while the latter breed originates from Rambouillet and Île-de-France (https://www.fondazioneslowfood.com/en/ark-of-taste-slow-food/precocious-merinos-goat/). The results of our ADMIXTURE analysis show that MP and MB animals as well as crossbreds, share some ancestry with the Swiss White Alpine, a breed which derives from a cross between the Swiss White Mountain and Île-de-France (http://afs.okstate.edu/breeds/sheep/swisswhitealpine). Indeed, whole-genome 600 K SNP array data confirm the close relationship of Rambouillet and Merino breeds from Spain and France (Rochus et al., 2020), also depicting a migration event consistent with gene flow from Rambouillet to Île-de-France breed (Rochus et al., 2018). The MP that once dominated in Portugal decreased in numbers in the second half of the 20th century, as black wool became less valuable, persisting in marginal areas and maintaining its identity with minimum influences from exotic stock.

Additionally, the phylogenetic analysis of sheep mitogenomes showed that Portuguese native breeds and two animals of the Akkaraman breed from Turkey are genetically close which provides support to a direct influence of Near-Eastern stock having reached the Iberian Peninsula *via* the Mediterranean dispersion routes (Zilhã, 2001; Pereira et al., 2006; Gron et al., 2020). Our results are in agreement with a study of 501 D-loop sequences representing 19 Iberian breeds in which haplogroup B also had the highest frequency (>98%) (Pedrosa et al., 2007). Consistently, a recent study of sheep mitogenomes also showed that haplogroup B had the highest frequency in Southwest Europe (>90%) (Lv et al., 2015). The clustering of three crossbreds with Italian Merino sheep (HM236174; HM236175; KF302440; KF302445; KF302446) could reflect recent upgrading. In future studies it would be extremely interesting to extend these analyses to other local breeds from the Iberian Peninsula, in particular the coarse-wool sheep (Churra) that are raised in this region in distinct environments from north to south (see Figure 1).

## 5 Conclusion

We examined the patterns of genomic diversity and population structure of four Portuguese native sheep within other Iberian and worldwide breeds. Our results suggest these Portuguese breeds are not genetically compromised, showing moderate diversity and negligible inbreeding. Expanding our study to a larger number of animals and farms should allow for more comprehensive inferences on Iberian sheep biodiversity to define management and conservation plans. The population structure analyses depicted the Iberian Merino sheep as a well differentiated breed group. The Merinos are thought to have been developed in the Iberian Peninsula and their selection for wool, meat and adaptive traits for local conditions appear to have resulted in a distinct genetic make-up. The gene flow between the Portuguese and Spanish Merino breeds depicted in the phylogenetic analysis could be explained by traditional transhumance routes which increase the chance for crossbreeding. Portuguese native breeds formed a tight clade within major haplogroup B in the phylogeny of sheep mitogenomes. This is the first study of Portuguese native sheep using whole genomes and sets the ground for defining ancestry informative SNPs for breed-specific admixture analysis, i.e., a powerful tool for breed assignment and traceability of certified breed-products, but also for genome-wide association studies. In addition, the genomic data we generated will be most valuable for a combined analysis of sheep genomes retrieved from historic and archaeological specimens to investigate the origins, evolution and modes of improvement of native Iberian sheep.

## Data Availability

The datasets presented in this study can be found in online repositories. The names of the repository/repositories and accession number(s) can be found in the article/Supplementary Material. The raw reads have been deposited in the Sequence Read Archive (SRR16085831 - SRR16085886) with the corresponding Bioproject PRJNA764662.

## Abbreviations

CAM, Campaniça; CB, Crossbred Sheep; DNA, Deoxyribonucleic acid; HTS, High-throughput sequencing; MAF, Minor allele frequency; MB, Merino Branco; ML, maximum likelihood; MP, Merino Preto; PCA, Principal component analysis; ROH, Runs of homozygosity; SE, Bordaleira Serra da Estrela; SNP, Single-nucleotide polymorphism.
