# Supplementary material for: Genome-wide assessment of the population structure and genetic diversity of four Portuguese native sheep breeds
Source: Front Genet. 2023 Jan 13;14:1109490. doi: 10.3389/fgene.2023.1109490 (PMC9880275; doi:10.3389/fgene.2023.1109490)
Supplement: Supplementary file 1 [file Table5.pdf]

Table S5: SNP distribution across genomic regions and functional effects

Location	Counts	Percentage
Exonic	209 884	0,7%
Intergenic	20 172 060	65,2%
Intronic	10 325 337	33,4%
UTR5	51 111	0,2%
UTR3	173 004	0,6%

Effect	Counts	Percentage
Nonsynonymous SNV	80 882	38,5%
Stopgain	880	0,4%
Stoploss	111	0,1%
Synonymous SNV	120 172	57,2%
Unknown**	7 870	3,7%

**"unknown" means that the gene structure is not correctly annotated (complete ORF information is not available).
