# Supplementary material for: Genome-wide assessment of the population structure and genetic diversity of four Portuguese native sheep breeds
Source: Front Genet. 2023 Jan 13;14:1109490. doi: 10.3389/fgene.2023.1109490 (PMC9880275; doi:10.3389/fgene.2023.1109490)
Supplement: Supplementary file 2 [file DataSheet2.pdf]

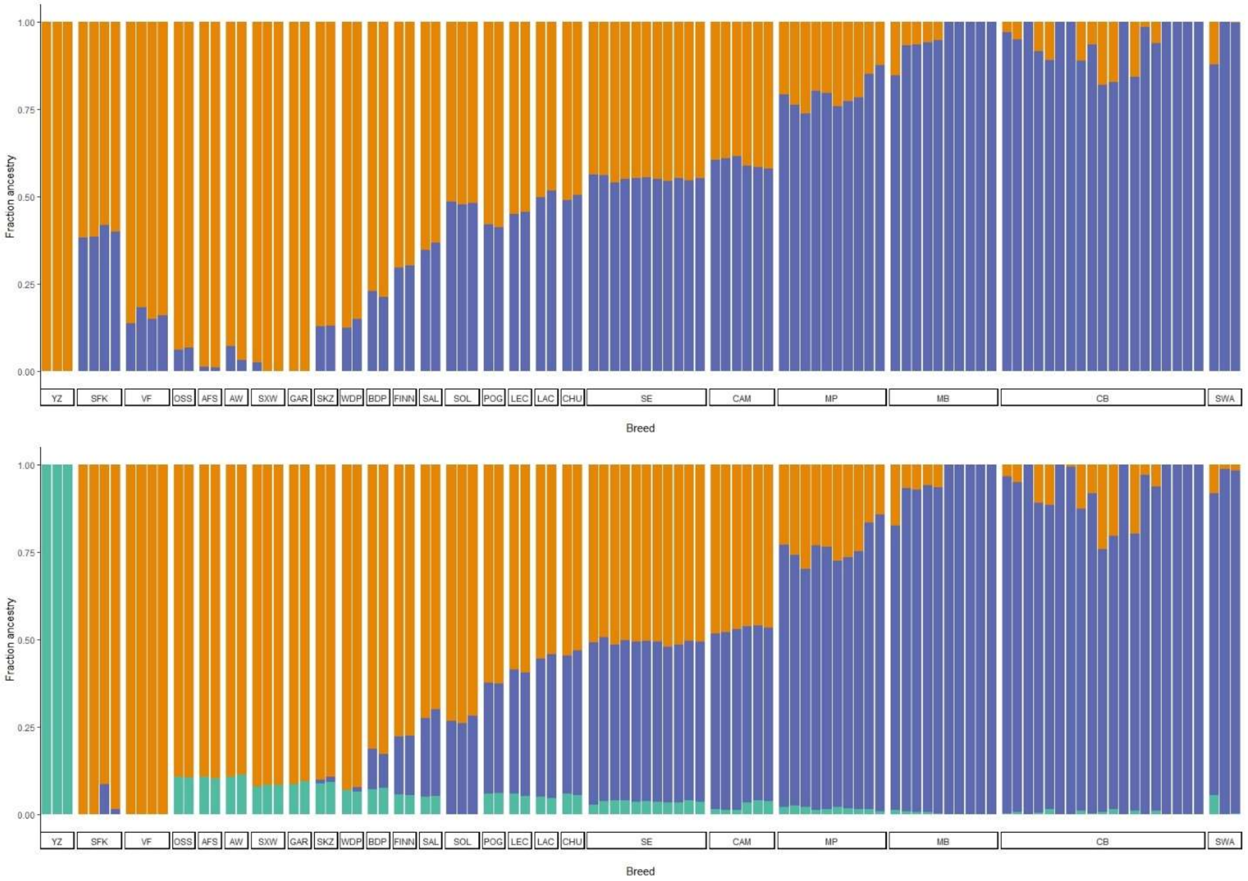
Fraction ancestry
1.00
0.75
0.50
0.25
0.00
YZ SFK VF OSS AFS AW SXW GAR SKZ WDP BDP FINN SAL SOL POG LEC LAC CHU SE CAM MP MB CB SWA
Breed
Fraction ancestry
1.00
0.75
0.50
0.25
0.00
YZ SFK VF OSS AFS AW SXW GAR SKZ WDP BDP FINN SAL SOL POG LEC LAC CHU SE CAM MP MB CB SWA
Breed

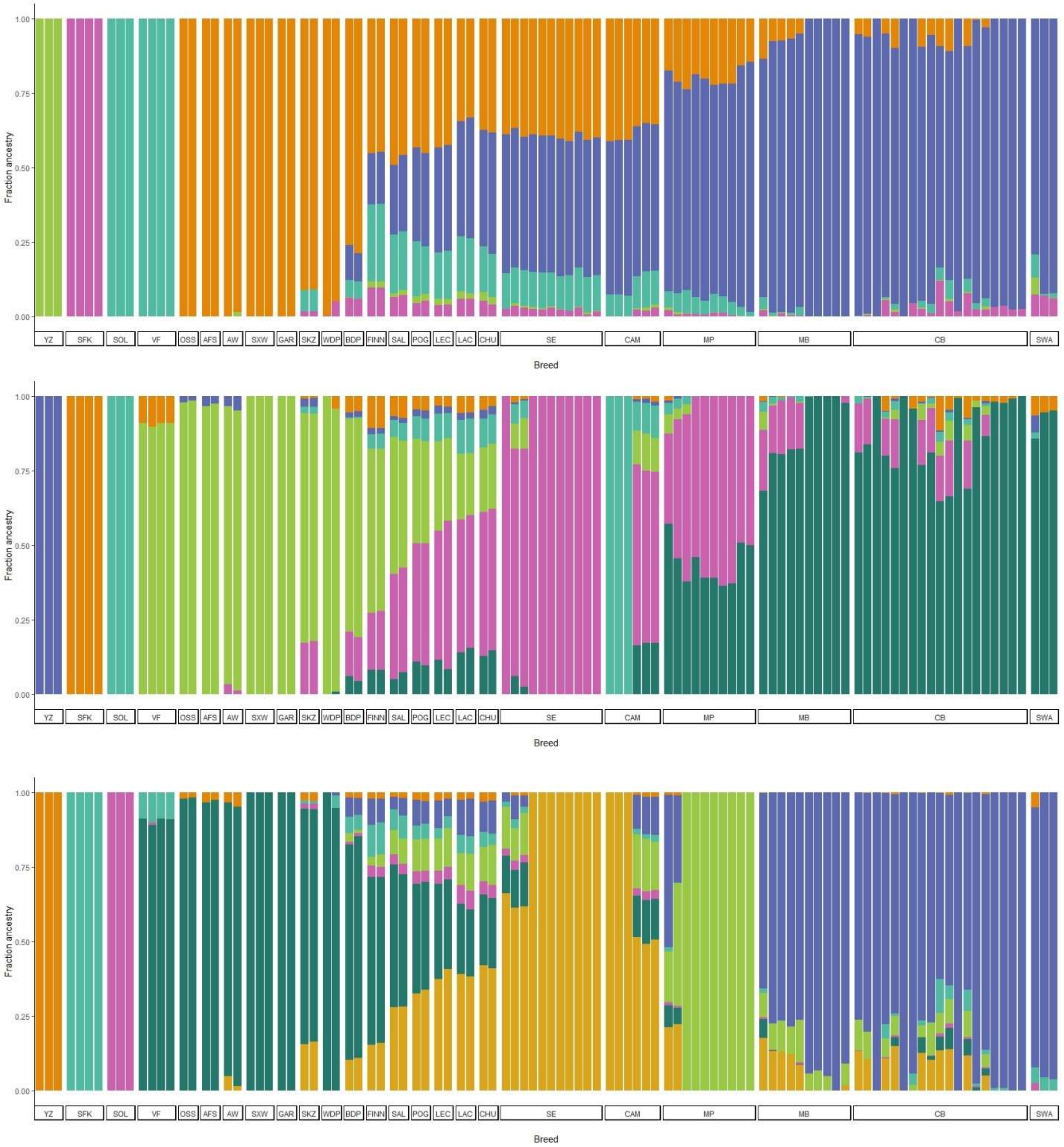
Fraction ancestry
1.00
0.75
0.50
0.25
0.00
YZ SFK SOL VF OSS AFS AW SXW GAR SKZ WDP BDP FINN SAL POG LEC LAC CHU SE CAM MP MB CB SWA
Breed

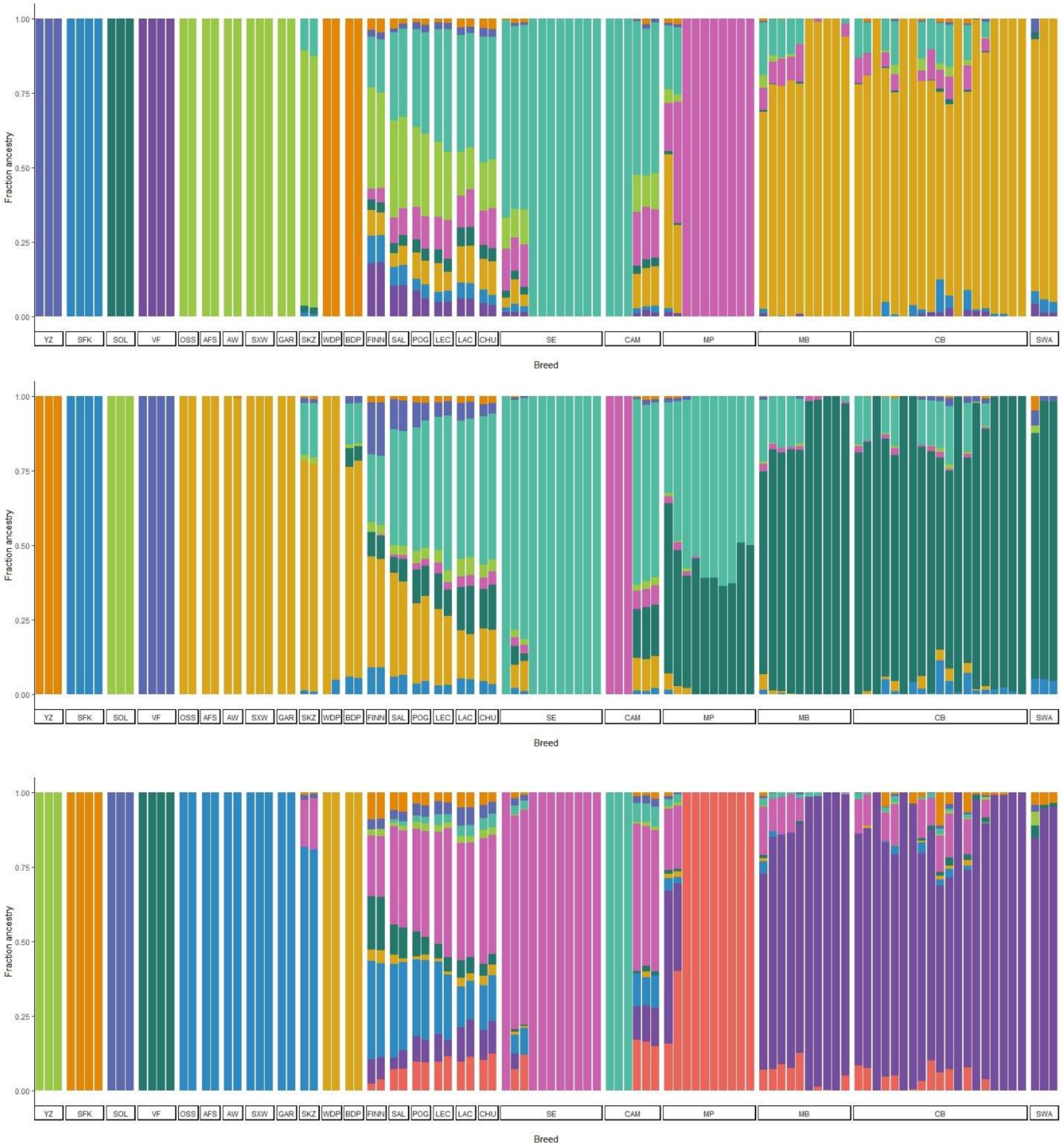

Fraction ancestry
1.00
0.75
0.50
0.25
0.00
YZ
SFK
SOL
VF
OSS
AFS
AW
SXW
GAR
SKZ
WDP
BDP
FINN
SAL
POG
LEC
LAC
CHU
SE
CAM
MP
MB
CB
SWA
Breed

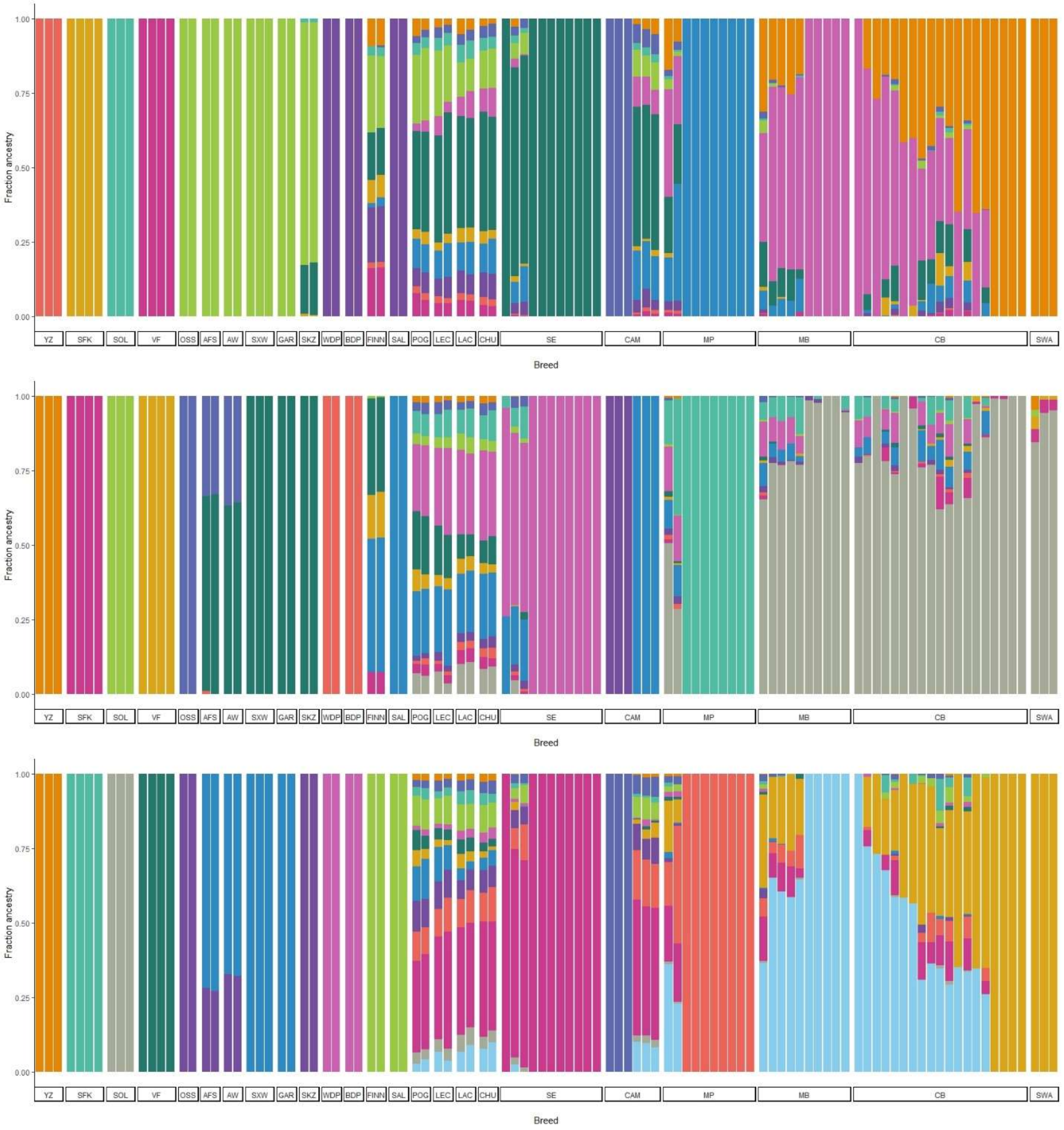

Fraction ancestry
0.00
0.25
0.50
0.75
1.00
YZ
SFK
SOL
VF
OSS
AFS
AW
SXW
GAR
SKZ
WDP
BDP
FINN
SAL
POG
LEC
LAC
CHU
SE
CAM
MP
MB
CB
SWA
Breed

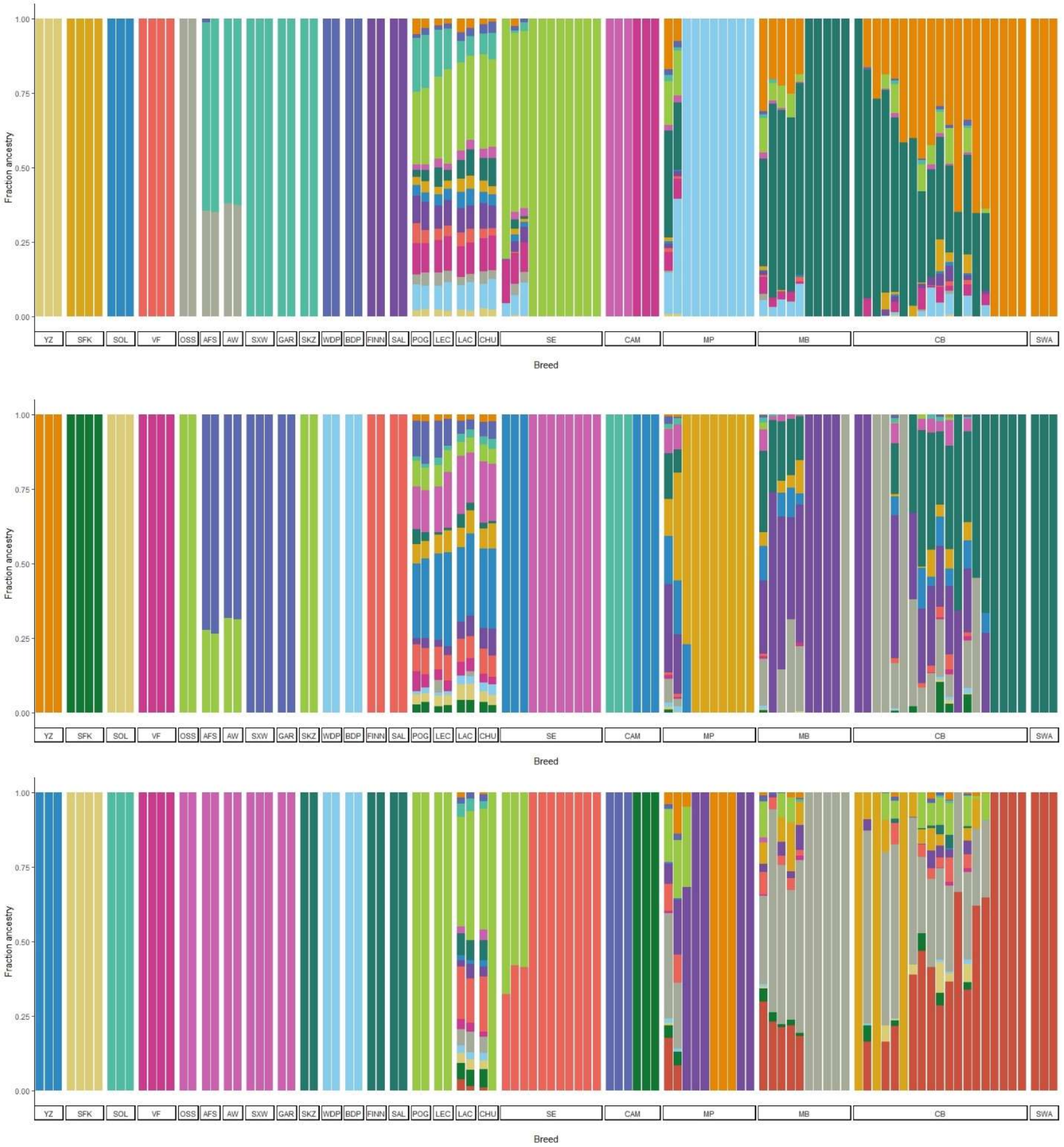
Fraction ancestry
1.00
0.75
0.50
0.25
0.00
YZ
SFK
SOL
VF
OSS
AFS
AW
SXW
GAR
SKZ
WDP
BDP
FINN
SAL
POG
LEC
LAC
CHU
SE
CAM
MP
MB
CB
SWA
Breed

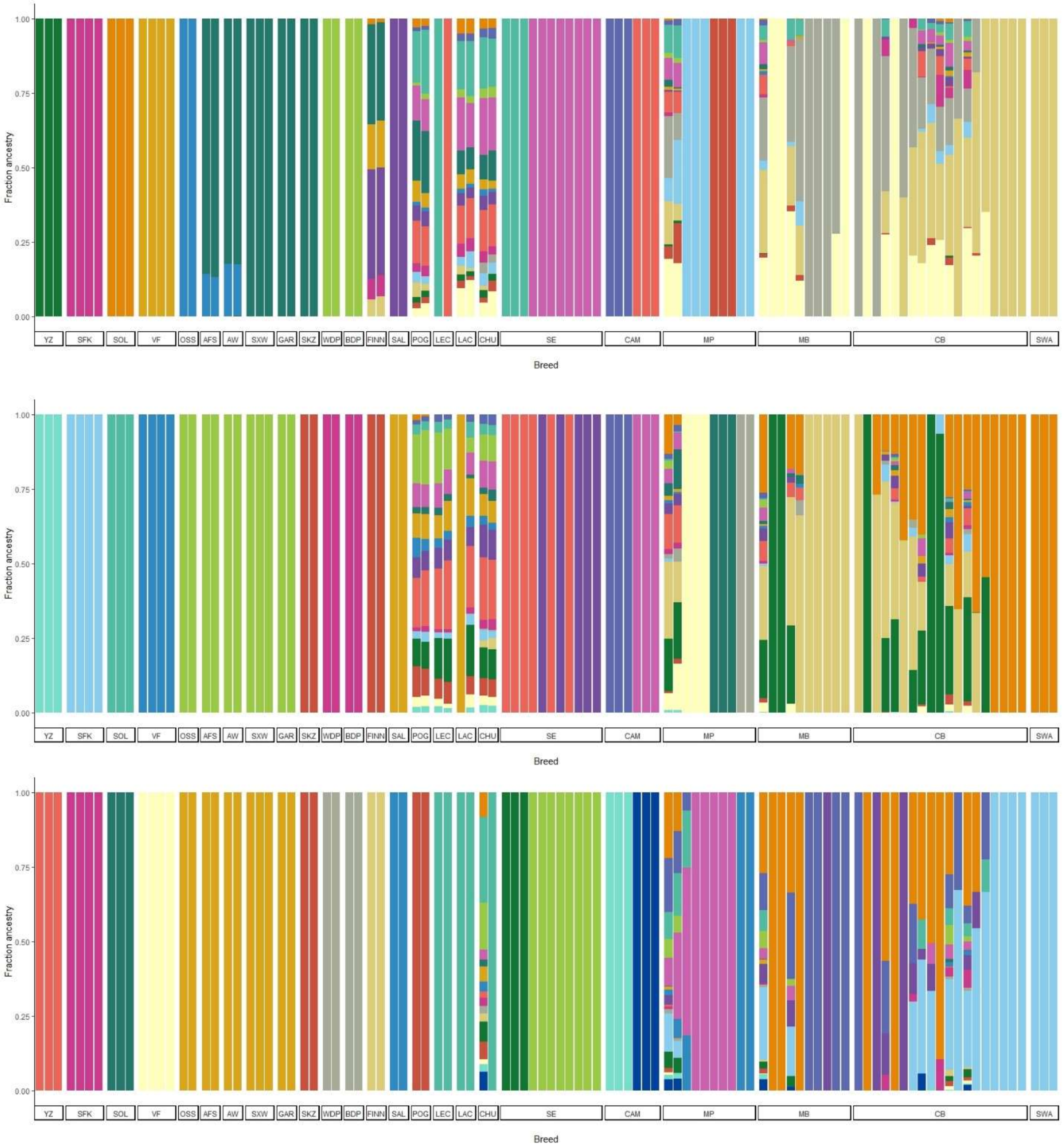

Fraction ancestry
1.00
0.75
0.50
0.25
0.00
YZ SFK SOL VF OSS AFS AW SXW GAR SKZ WDP BDP FINN SAL POG LEC LAC CHU SE CAM MP MB CB SWA
Breed
Fraction ancestry
1.00
0.75
0.50
0.25
0.00
YZ SFK SOL VF OSS AFS AW SXW GAR SKZ WDP BDP FINN SAL POG LEC LAC CHU SE CAM MP MB CB SWA
Breed
Fraction ancestry
1.00
0.75
0.50
0.25
0.00
YZ SFK SOL VF OSS AFS AW SXW GAR SKZ WDP BDP FINN SAL POG LEC LAC CHU SE CAM MP MB CB SWA
Breed

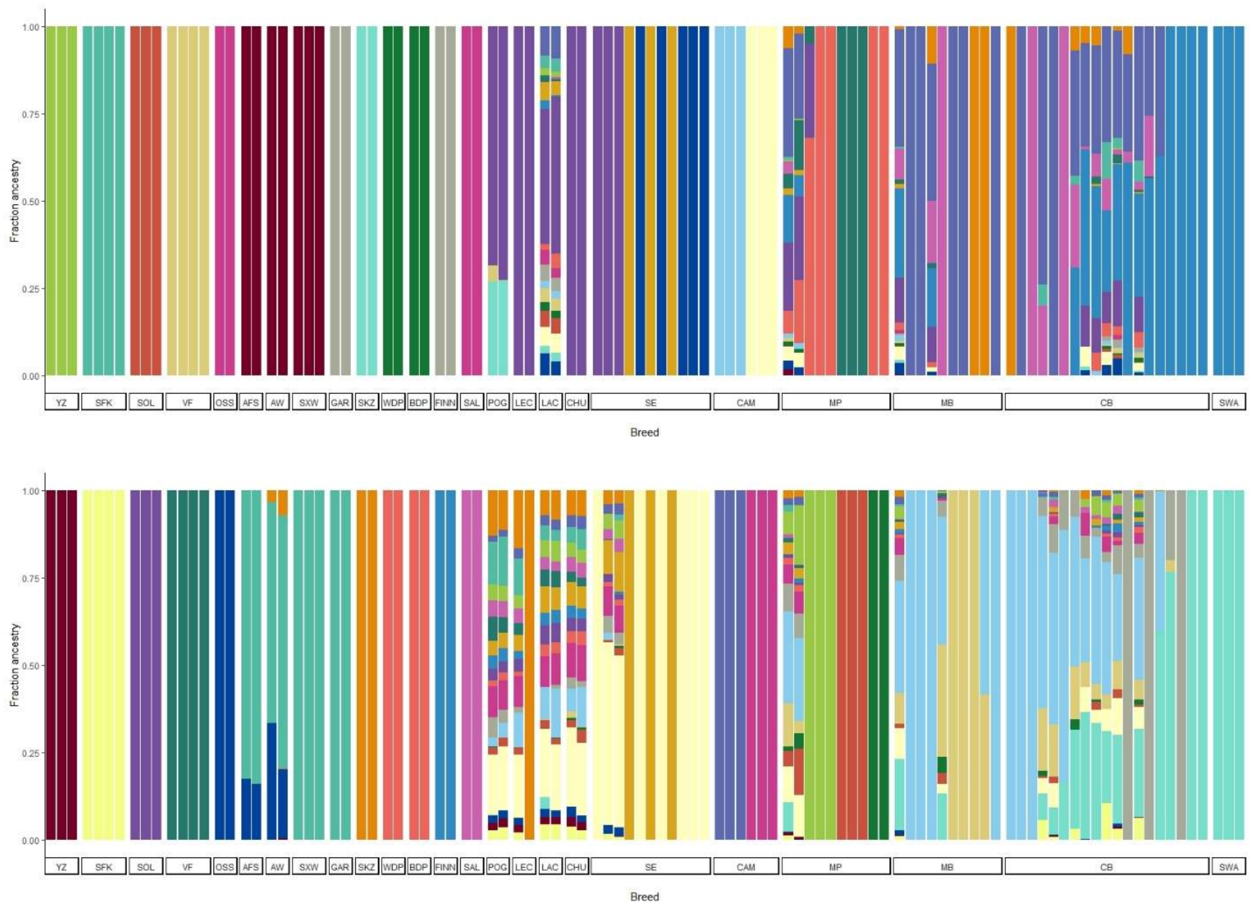

Supplementary File S2: Model-based clustering analysis of Iberian and worldwide sheep. The proportions of the inferred ancestral clusters are depicted by the different colours for K=2 to K=21 with each individual represented by a bar and sorted by breed. CAM—Campaniça; MB—Merino Branco; MP—Merino Preto; SE—Serra da Estrela; and CB—Crossbreds.
