# Supplementary material for: Genome-wide assessment of the population structure and genetic diversity of four Portuguese native sheep breeds
Source: Front Genet. 2023 Jan 13;14:1109490. doi: 10.3389/fgene.2023.1109490 (PMC9880275; doi:10.3389/fgene.2023.1109490)
Supplement: Supplementary file 3 [file Table4.pdf]

Table S4: Sheep mitogenomes included in the analysis

GenBank reference	Species/Breed	Sampling Location	Haplogroup	Reference
HM236177	Karakas	Turkey	B1a	[31]
NC001941	Merinolandschaf	N.A.	B1a	
HM236176	Karakas	Turkey	B1a	
EF490452	Finn Dorset	N.A.	B1a	
EF490456	Finn Dorset	N.A.	B1a	
KF938341	Udmurtian local	Russia	B1a	
KF938358	Kulunda	Russia	B1a	
KF302447	Lacaune	France	B1a	
KF302453	Lacaune	France	B1a	
KF302448	Gentile di Puglia	Italy	B1a	
KF302452	Comisana	Italy	B1a	
KF302449	Sopravissana	Italy	B1a	
KF302450	Appenninica	Italy	B1a	
KF938359	Oxford Down	United Kingdom	B1a	
KF302454	Sopravissana	Italy	B1a	
KF302455	Gentile di Puglia	Italy	B1a	
EF490453	Finn Dorset	N.A.	B1a	
EF490454	Finn Dorset	N.A.	B1a	
HM236184	*Ovis musimon*	Germany	N.A.	
HM236185	*Ovis musimon*	Germany	N.A.	
KF938328	Jingzhong	China	B1a	
KF302460	Lancaune	France	B1a	
KF302461	Comisana	Italy	B1a	
KF302462	Comisana	Italy	B1a	
KF302456	Sopravissana	Italy	B1a	
KF302458	Sopravissana	Italy	B1a	
KF938344	Mazekh	Azerbaijan	B1a	
KF938343	Tushin	Russia	B1a	
KF938333	Kazakh	China	B1b	
KF938335	Lanzhou Large-taile	China	B2	
KF938325	Qinhai Tibetan	China	A2	
KF938334	Kirghiz	China	A2	
KF938321	Baerchuke	China	A1b	
KF938319	Ujimqin	China	A1b	
HM236174	Merino	Australia	A1a	
KF302440	Comisana	Italy	A1a	
KF302445	Comisana	Italy	A1a	
KF302446	Merinizzata Italiana	Italy	A1a	
HM236175	Merino	Australia	A1a	
HM236180	Morkaraman	Turkey	D	
HM236181	Morkaraman	Turkey	D	
HM236178	Karakas	Turkey	C	

Table S4: Sheep mitogenomes included in the analysis

GenBank accession nº	Species/Breed	Sampling Location	Haplogroup	Reference
HM236179	Morkaraman	Turkey	C	[31]
HM236182	Awassi	Israel	E	
HM236183	Tuj	Turkey	E	
HM236186	*Ovis vignei*	Kazakhstan	N.A.	
HM236189	*Ovis vignei*	Kazakhstan	N.A.	
HM236188	*Ovis ammon*	Kazakhstan	N.A.	
