# Supplementary material for: Genome-wide assessment of the population structure and genetic diversity of four Portuguese native sheep breeds
Source: Front Genet. 2023 Jan 13;14:1109490. doi: 10.3389/fgene.2023.1109490 (PMC9880275; doi:10.3389/fgene.2023.1109490)
Supplement: Supplementary file 4 [file Table1.pdf]

Table S1: Details on locations, breed characteristics, sequencing statistics and accession numbers

Breed	Sample name	Location	Number of flocks	Wool type	Main purpose	sex	Number of raw read pairs	Average depth	NCBI Biosample	Run Reference	NCBI Bioproject	Data Sources
Campaniça	Cam_C11	Serpa	2	Medium-fine	meat	M	315468173	22,46	SAMN21521677	SRR16085886	PRJNA764662	This study
Campaniça	Cam_C12	Serpa		Medium-fine	meat	M	316013003	22,50	SAMN21521678	SRR16085885	PRJNA764662	This study
Campaniça	Cam_C13	Serpa		Medium-fine	meat	M	314369703	22,38	SAMN21521679	SRR16085874	PRJNA764662	This study
Campaniça	Cam_C15	Mértola		Medium-fine	meat	M	312002762	22,21	SAMN21521680	SRR16085863	PRJNA764662	This study
Campaniça	Cam_C16	Mértola		Medium-fine	meat	M	313775775	22,34	SAMN21521681	SRR16085852	PRJNA764662	This study
Campaniça	Cam_C17	Mértola		Medium-fine	meat	M	310997092	22,14	SAMN21521682	SRR16085841	PRJNA764662	This study
Merino Branco	MB_C24	Serpa	4	Merino	meat	F	316277616	22,52	SAMN21521683	SRR16085834	PRJNA764662	This study
Merino Branco	MB_C25	Serpa		Merino	meat	F	313836083	22,34	SAMN21521684	SRR16085833	PRJNA764662	This study
Merino Branco	MB_C88	Serpa		Merino	meat	F	312206781	22,23	SAMN21521685	SRR16085832	PRJNA764662	This study
Merino Branco	MB_J34	Portalegre		Merino	meat	F	293086223	20,87	SAMN21521686	SRR16085831	PRJNA764662	This study
Merino Branco	MB_J42	Portalegre		Merino	meat	F	311776762	22,20	SAMN21521687	SRR16085884	PRJNA764662	This study
Merino Branco	MB_M105	Portalegre		Merino	meat	F	313751466	22,34	SAMN21521688	SRR16085883	PRJNA764662	This study
Merino Branco	MB_M25	Portalegre		Merino	meat	F	312717085	22,27	SAMN21521689	SRR16085882	PRJNA764662	This study
Merino Branco	MB_M82	Portalegre		Merino	meat	F	315245190	22,45	SAMN21521690	SRR16085881	PRJNA764662	This study
Merino Branco	MB_Q25	Moura		Merino	meat	F	312390522	22,24	SAMN21521691	SRR16085880	PRJNA764662	This study
Merino Branco	MB_Q3	Moura		Merino	meat	F	313416622	22,31	SAMN21521692	SRR16085879	PRJNA764662	This study
Crossbred	MC_A12	Castro Verde	10	Merino	meat	F	310045172	22,07	SAMN21521693	SRR16085878	PRJNA764662	This study
Crossbred	MC_A36	Castro Verde		Merino	meat	F	308044914	21,93	SAMN21521694	SRR16085877	PRJNA764662	This study
Crossbred	MC_B22	Évora		Merino	meat	F	315087930	22,43	SAMN21521695	SRR16085876	PRJNA764662	This study
Crossbred	MC_B6	Évora		Merino	meat	F	312206720	22,23	SAMN21521696	SRR16085875	PRJNA764662	This study
Crossbred	MC_E44	Almodôvar		Merino	meat	F	313596898	22,33	SAMN21521697	SRR16085873	PRJNA764662	This study
Crossbred	MC_E73	Almodôvar		Merino	meat	F	313797457	22,34	SAMN21521698	SRR16085872	PRJNA764662	This study
Crossbred	MC_F84	Portel		Merino	meat	F	316356585	22,52	SAMN21521699	SRR16085871	PRJNA764662	This study
Crossbred	MC_F92	Portel		Merino	meat	F	317275202	22,59	SAMN21521700	SRR16085870	PRJNA764662	This study
Crossbred	MC_H73	Montemor-o-Novo		Merino	meat	F	310031472	22,07	SAMN21521701	SRR16085869	PRJNA764662	This study
Crossbred	MC_H74	Montemor-o-Novo		Merino	meat	F	314539661	22,39	SAMN21521702	SRR16085868	PRJNA764662	This study
Crossbred	MC_K17	Ferreira do Alentejo		Merino	meat	F	310031858	22,07	SAMN21521703	SRR16085867	PRJNA764662	This study
Crossbred	MC_K90	Ferreira do Alentejo		Merino	meat	F	314161623	22,37	SAMN21521704	SRR16085866	PRJNA764662	This study
Crossbred	MC_L3	Alvito		Merino	meat	F	314967190	22,43	SAMN21521705	SRR16085865	PRJNA764662	This study
Crossbred	MC_N31	Évora		Merino	meat	F	313543960	22,32	SAMN21521706	SRR16085864	PRJNA764662	This study
Crossbred	MC_N8	Évora		Merino	meat	F	316588954	22,54	SAMN21521707	SRR16085862	PRJNA764662	This study

Table S1: Details on locations, breed characteristics, sequencing statistics and accession numbers

Breed	Sample name	Location	Number of flocks	Wool type	Main purpose	sex	Number of raw read pairs	Average depth	NCBI Biosample	Run Reference	NCBI Bioproject	Data Sources
Crossbred	MC_O102	Odemira		Merino	meat	F	312674891	22,26	SAMN21521708	SRR16085861	PRJNA764662	This study
Crossbred	MC_O59	Odemira		Merino	meat	F	313006625	22,29	SAMN21521709	SRR16085860	PRJNA764662	This study
Crossbred	MC_P66	Ponte de Sor		Merino	meat	F	317496111	22,61	SAMN21521710	SRR16085859	PRJNA764662	This study
Crossbred	MC_P95	Ponte de Sor		Merino	meat	F	317665010	22,62	SAMN21521711	SRR16085858	PRJNA764662	This study
Merino Preto	MP_D34	Serpa		Merino	meat	F	312820197	22,27	SAMN21521712	SRR16085857	PRJNA764662	This study
Merino Preto	MP_D4	Serpa		Merino	meat	F	313809720	22,34	SAMN21521713	SRR16085856	PRJNA764662	This study
Merino Preto	MP_D54	Serpa		Merino	meat	F	317185295	22,58	SAMN21521715	SRR16085854	PRJNA764662	This study
Merino Preto	MP_D5	Serpa		Merino	meat	F	313915649	22,35	SAMN21521714	SRR16085855	PRJNA764662	This study
Merino Preto	MP_D82	Serpa		Merino	meat	F	312725574	22,27	SAMN21521716	SRR16085853	PRJNA764662	This study
Merino Preto	MP_J11	Portalegre	3	Merino	meat	F	313037973	22,29	SAMN21521717	SRR16085851	PRJNA764662	This study
Merino Preto	MP_J27	Portalegre		Merino	meat	F	315488068	22,46	SAMN21521718	SRR16085850	PRJNA764662	This study
Merino Preto	MP_Q107	Moura		Merino	meat	F	314141424	22,37	SAMN21521719	SRR16085849	PRJNA764662	This study
Merino Preto	MP_Q67	Moura		Merino	meat	F	315412081	22,46	SAMN21521720	SRR16085848	PRJNA764662	This study
Merino Preto	MP_Q75	Moura		Merino	meat	F	264657927	18,84	SAMN21521721	SRR16085847	PRJNA764662	This study
Bordaleira Serra da Estrela	Seb1	Nelas		Medium-fine	dairy	F	299836265	21,35	SAMN21521728	SRR16085839	PRJNA764662	This study
Bordaleira Serra da Estrela	SE2	Nelas		Medium-fine	dairy	M	311492532	22,18	SAMN21521722	SRR16085845	PRJNA764662	This study
Bordaleira Serra da Estrela	Seb4A	Celorico da Beira		Medium-fine	dairy	M	314605245	22,40	SAMN21521732	SRR16085835	PRJNA764662	This study
Bordaleira Serra da Estrela	SE2A	Celorico da Beira		Medium-fine	dairy	M	312861502	22,28	SAMN21521723	SRR16085846	PRJNA764662	This study
Bordaleira Serra da Estrela	Seb30	Oliveira do Hospital		Medium-fine	dairy	M	314589451	22,40	SAMN21521729	SRR16085838	PRJNA764662	This study
Bordaleira Serra da Estrela	Seb31	Oliveira do Hospital	9	Medium-fine	dairy	F	316308978	22,52	SAMN21521730	SRR16085837	PRJNA764662	This study
Bordaleira Serra da Estrela	Seb32	Seia		Medium-fine	dairy	M	313945643	22,35	SAMN21521731	SRR16085836	PRJNA764662	This study
Bordaleira Serra da Estrela	SE7A	Seia		Medium-fine	dairy	M	314399526	22,38	SAMN21521726	SRR16085842	PRJNA764662	This study
Bordaleira Serra da Estrela	SE9	Gouveia		Medium-fine	dairy	M	314658926	22,40	SAMN21521727	SRR16085840	PRJNA764662	This study
Bordaleira Serra da Estrela	SE5A	Gouveia		Medium-fine	dairy	M	311642566	22,19	SAMN21521725	SRR16085843	PRJNA764662	This study
Bordaleira Serra da Estrela	SE3	Carregal do Sal		Medium-fine	dairy	M	312298448	22,24	SAMN21521724	SRR16085844	PRJNA764662	This study
