# Supplementary material for: Genome-wide assessment of the population structure and genetic diversity of four Portuguese native sheep breeds
Source: Front Genet. 2023 Jan 13;14:1109490. doi: 10.3389/fgene.2023.1109490 (PMC9880275; doi:10.3389/fgene.2023.1109490)
Supplement: Supplementary file 5 [file Table2.pdf]

Table S2: Worldwide sheep genomes included in the analysis.

NCBI Project	Sample name	NCBI Biosample	Run reference	Species	Breed	Region	Raw reads	Average Depth
PRJNA624020	FINN317	SAMN14590314	SRR11657543	Ovis aries	Finnsheep	Finland	600,487,082	28.87
PRJNA624020	FINN307	SAMN14590310	SRR11657547	Ovis aries	Finnsheep	Finland	602,566,288	28.81
PRJNA624020	YZ.9	SAMN14590397	SRR11657502	Ovis orientalis	Asiatic Mouflon	Iran	558,995,366	24.93
PRJNA624020	YZ.11	SAMN14590399	SRR11657504	Ovis orientalis	Asiatic Mouflon	Iran	813,551,536	36.93
PRJNA624020	YZ.12	SAMN14590400	SRR11657505	Ovis orientalis	Asiatic Mouflon	Iran	628,617,440	28.71
PRJNA624020	WDP155	SAMN14590249	SRR11657614	Ovis aries	Dorper (white head)	South Africa	527,977,370	25.43
PRJNA624020	WDP153	SAMN14590248	SRR11657613	Ovis aries	Dorper (white head)	South Africa	555,838,114	26.48
PRJNA624020	BDP357	SAMN14590250	SRR11657527	Ovis aries	Dorper (black head)	South Africa	562,463,000	26.70
PRJNA624020	BDP370	SAMN14590251	SRR11657526	Ovis aries	Dorper (black head)	South Africa	527,291,888	25.33
PRJNA624020	SFK193	SAMN14590275	SRR11657657	Ovis aries	Suffolk	England	520,722,616	25.62
PRJNA624020	SFK194	SAMN14590276	SRR11657656	Ovis aries	Suffolk	England	557,767,122	27.74
PRJNA624020	SFK195	SAMN14590277	SRR11657655	Ovis aries	Suffolk	England	618,986,928	28.63
PRJNA624020	SFK197	SAMN14590278	SRR11657654	Ovis aries	Suffolk	England	537,817,002	24.95
PRJNA624020	SXW16	SAMN14590155	SRR11657682	Ovis aries	Small-tailed Han	China	525,422,520	24.99
PRJNA624020	SXW17	SAMN14590156	SRR11657681	Ovis aries	Small-tailed Han	China	588,281,912	28.44
PRJNA624020	SXW19	SAMN14590157	SRR11657603	Ovis aries	Small-tailed Han	China	580,668,804	27.85
PRJNA624020	VF-02	SAMN14590221	SRR11657482	Ovis aries	East Friesian	Germany	549,132,510	25.40
PRJNA624020	VF-06	SAMN14590222	SRR11657483	Ovis aries	East Friesian	Germany	534,819,868	24.85
PRJNA624020	VF-10	SAMN14590223	SRR11657484	Ovis aries	East Friesian	Germany	623,745,384	28.46
PRJNA624020	VF-12	SAMN14590224	SRR11657485	Ovis aries	East Friesian	Germany	561,116,650	25.88
PRJNA624020	Solognote24727	SAMN14590332	SRR11657711	Ovis aries	Solognote	France	531,568,510	25.43
PRJNA624020	Solognote24728	SAMN14590333	SRR11657710	Ovis aries	Solognote	France	561,963,204	26.65
PRJNA624020	Solognote24729	SAMN14590334	SRR11657709	Ovis aries	Solognote	France	600,131,468	27.79
PRJNA160933	CHU1	SAMN01000796	SRR501848	Ovis aries	Churra	Spain	359,853,850	25,08
PRJNA160933	CHU2	SAMN01000798	SRR501909	Ovis aries	Churra	Spain	407,016,366	28,37
PRJNA160933	AFS32	SAMN01000753	SRR501853	Ovis aries	Afshari	Israel	348,659,838	24,30

Table S2: Worldwide sheep genomes included in the analysis.

NCBI Project	Sample name	NCBI Biosample	Run reference	Species	Breed	Region	Raw reads	Average Depth
PRJNA160933	AFS33	SAMN01000771	SRR501871	Ovis aries	Afshari	Israel	381,271,154	26,57
PRJNA160933	GAR14	SAMN01000803	SRR501902	Ovis aries	Indian Garole	India	461,874,464	32,19
PRJNA160933	GAR4	SAMN01000804	SRR501903	Ovis aries	Indian Garole	India	403,622,222	28,13
PRJNA160933	MERA1	SAMN01000788	SRR501887	Ovis aries	Australian Merino	Australia	386,414,710	26,93
PRJNA160933	MER454	SAMN01000752	SRR501852	Ovis aries	Australian Merino	Australia	404,630,334	28,20
PRJNA160933	MERC1	SAMN01000768	SRR501868	Ovis aries	Australian Merino	Australia	385,542,370	26,87
PRJNA160933	LAC1	SAMN01000750	SRR501850	Ovis aries	Lacaune	France	456,072,640	31,79
PRJNA160933	LAC84	SAMN01000751	SRR501851	Ovis aries	Lacaune	France	377,546,128	26,31
PRJNA160933	SWAA27	SAMN01000780	SRR501884	Ovis aries	Swiss White Alpine	Switzerland	411,703,968	28,70
PRJNA160933	SWAN3	SAMN01000800	SRR501908	Ovis aries	Swiss White Alpine	Switzerland	407 525 606	28,40
PRJNA624020	AWA21	SAMN14590377	SRR11657624	Ovis aries	Awassi	Iraq	412,186,220	19.07
PRJNA160933	AWT1	SAMN01000794	SRR501893	Ovis aries	Turkish Awassi	Turkey	297,235,410	10.35
PRJNA160933	SKZ1	SAMN01000743	SRR501843	Ovis aries	Sakiz	Turkey	430,845,142	30.03
PRJNA160933	SKZ4	SAMN01000779	SRR501878	Ovis aries	Sakiz	Turkey	352,638,546	24.58
PRJNA160933	SALC1	SAMN01000741	SRR501841	Ovis aries	Salz	Spain	439,926,294	30.66
PRJNA160933	SALA2	SAMN01000742	SRR501842	Ovis aries	Salz	Spain	441,368,154	30.76
PRJNA645671	18	SAMN15517460	SRR12396863	Ovis aries	Ossimi	Egypt	469,720,032	49.11
PRJNA645671	19	SAMN15517461	SRR12396862	Ovis aries	Ossimi	Egypt	446,480,930	46.68
PRJNA645671	POG14	SAMN15517559	SRR12396864	Ovis aries	Pag Island sheep	Croatia	473,603,924	49.51
PRJNA645671	POG24	SAMN15517560	SRR12396860	Ovis aries	Pag Island sheep	Croatia	480,784,212	50.26
PRJNA645671	LEC9	SAMN15517575	SRR12396900	Ovis aries	Leccese	Italy	480,176,518	50.20
PRJNA645671	LEC12	SAMN15517574	SRR12396901	Ovis aries	Leccese	Italy	448,798,318	46.92
