# Supplementary figures and images for: Genome-wide assessment of the population structure and genetic diversity of four Portuguese native sheep breeds
Source: Front Genet. 2023 Jan 13;14:1109490. doi: 10.3389/fgene.2023.1109490 (PMC9880275; doi:10.3389/fgene.2023.1109490)

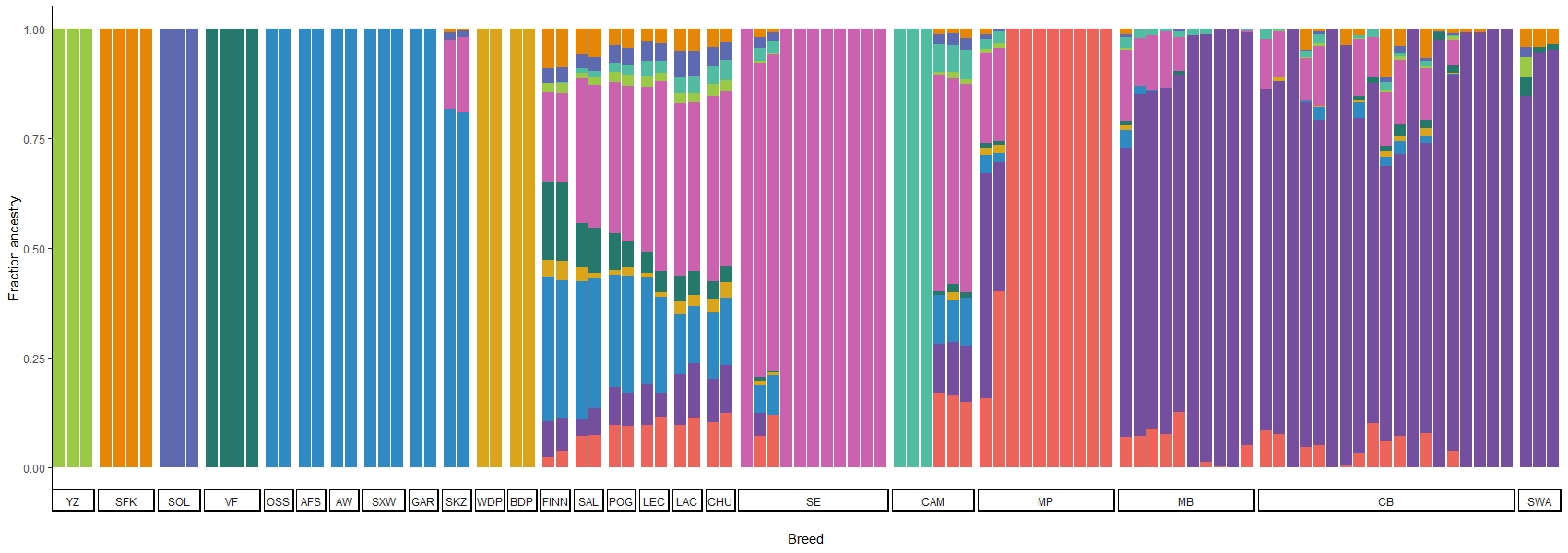

Supplement: Supplementary file 6 [file DataSheet1.ZIP › k10.jpeg]

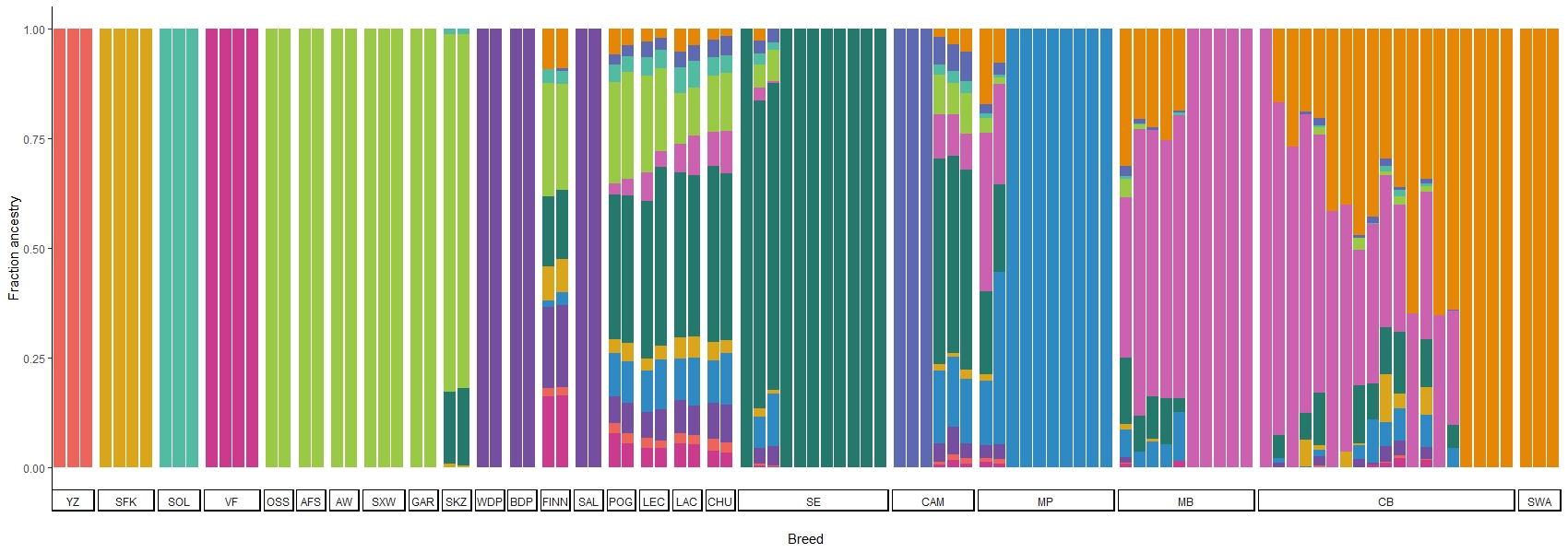

Supplement: Supplementary file 6 [file DataSheet1.ZIP › k11.jpeg]

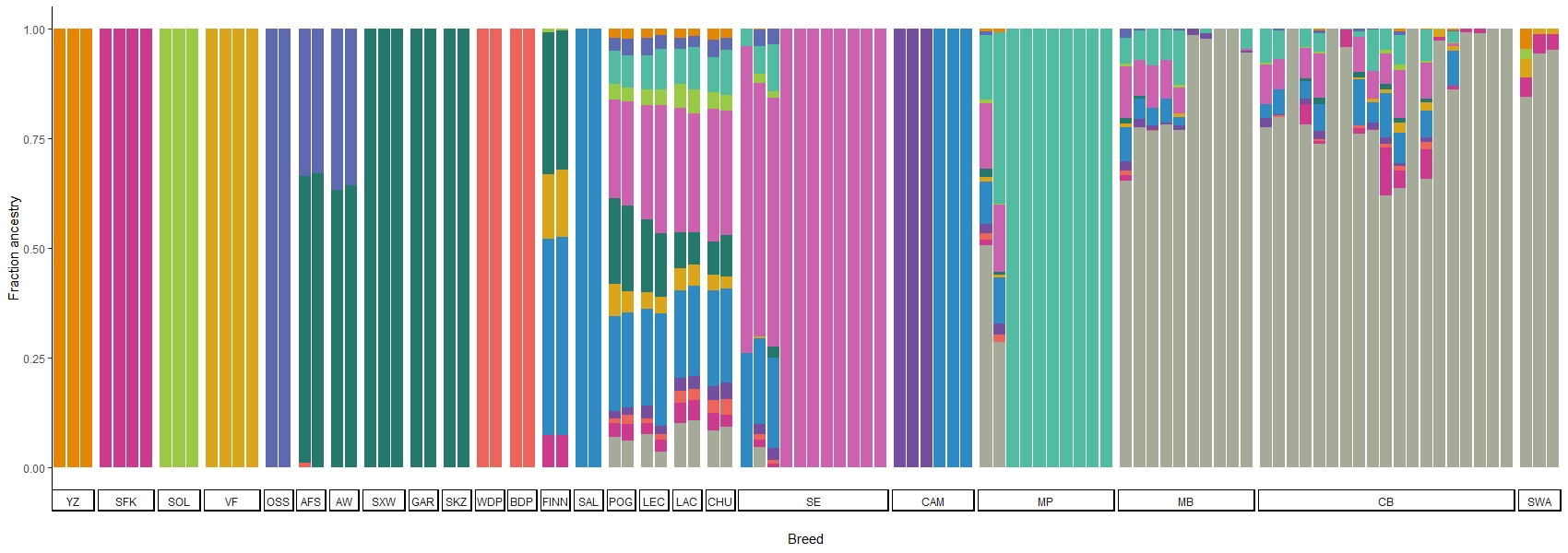

Supplement: Supplementary file 6 [file DataSheet1.ZIP › k12.jpeg]

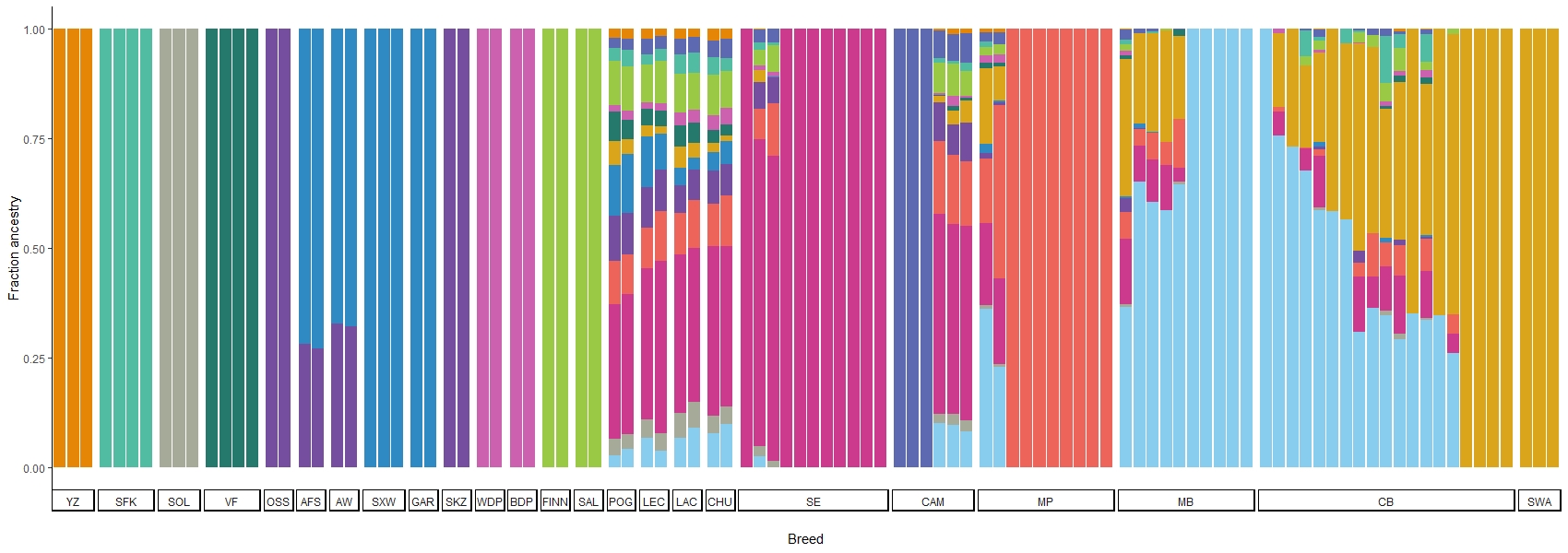

Supplement: Supplementary file 6 [file DataSheet1.ZIP › k13.jpeg]

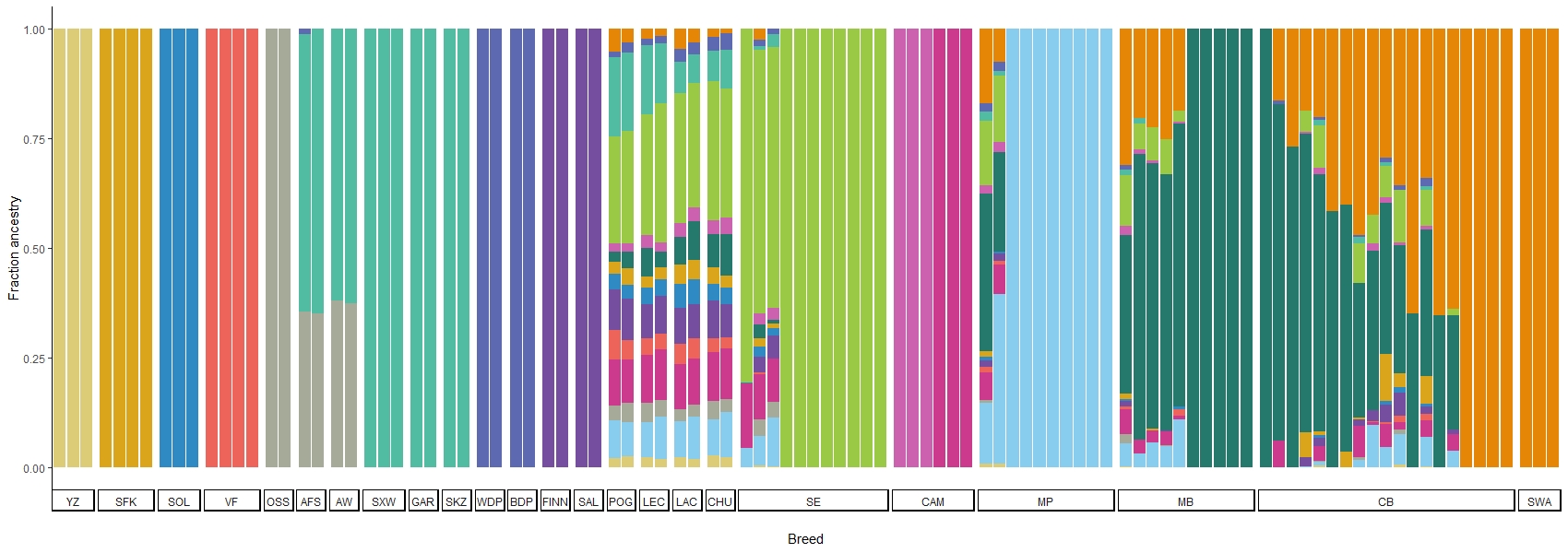

Supplement: Supplementary file 6 [file DataSheet1.ZIP › k14.jpeg]

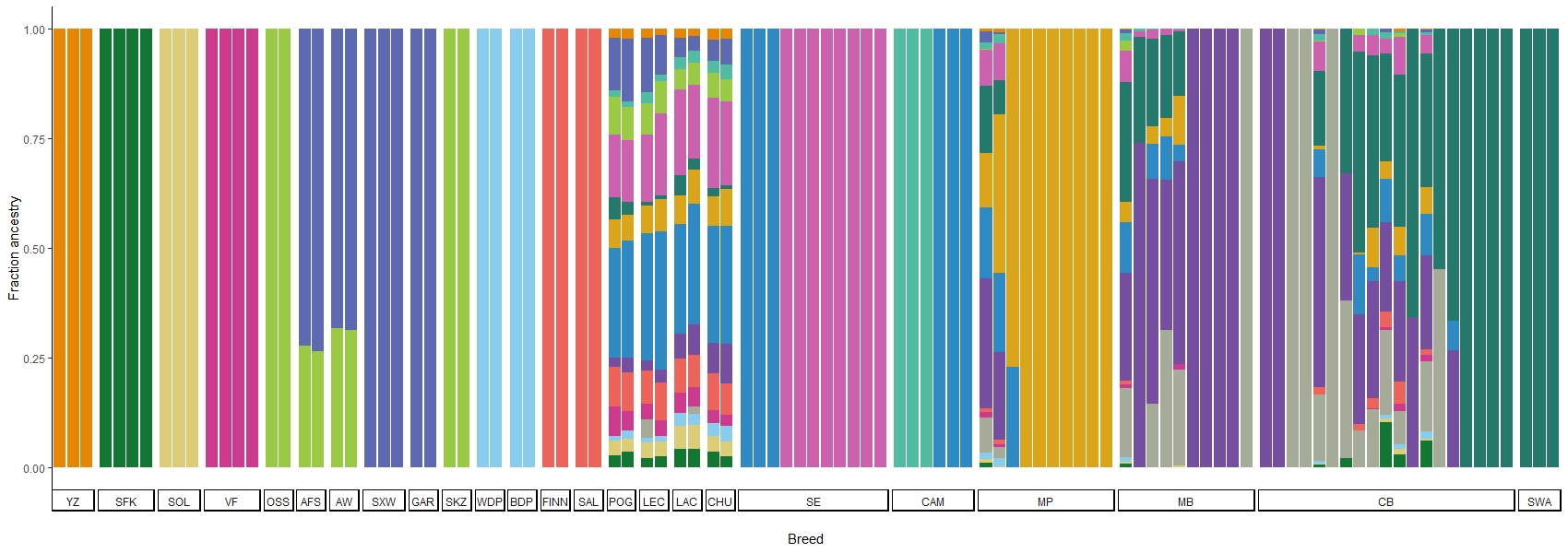

Supplement: Supplementary file 6 [file DataSheet1.ZIP › k15.jpeg]

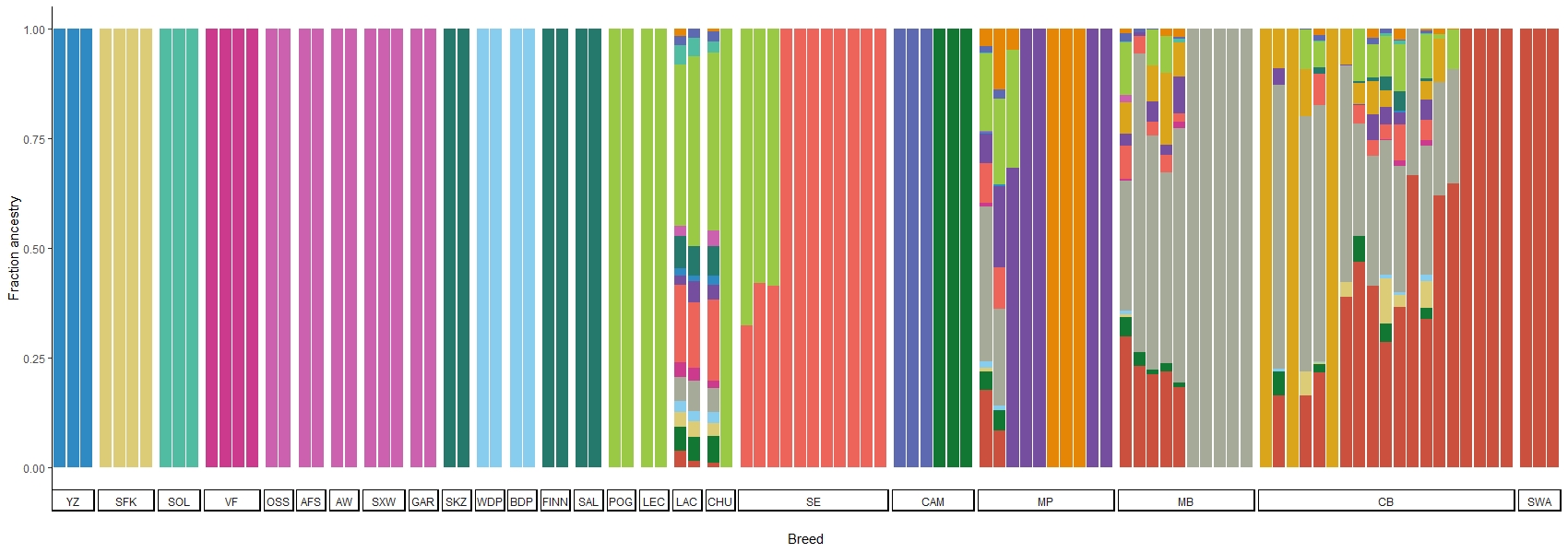

Supplement: Supplementary file 6 [file DataSheet1.ZIP › k16.jpeg]

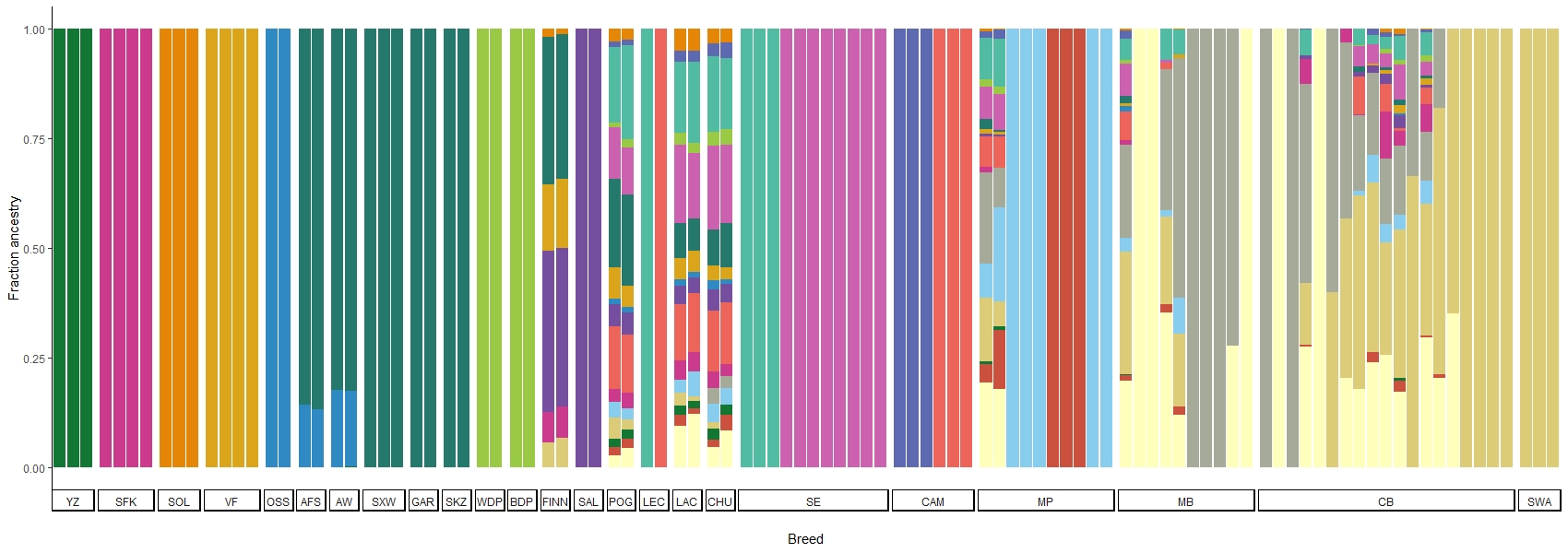

Supplement: Supplementary file 6 [file DataSheet1.ZIP › k17.jpeg]

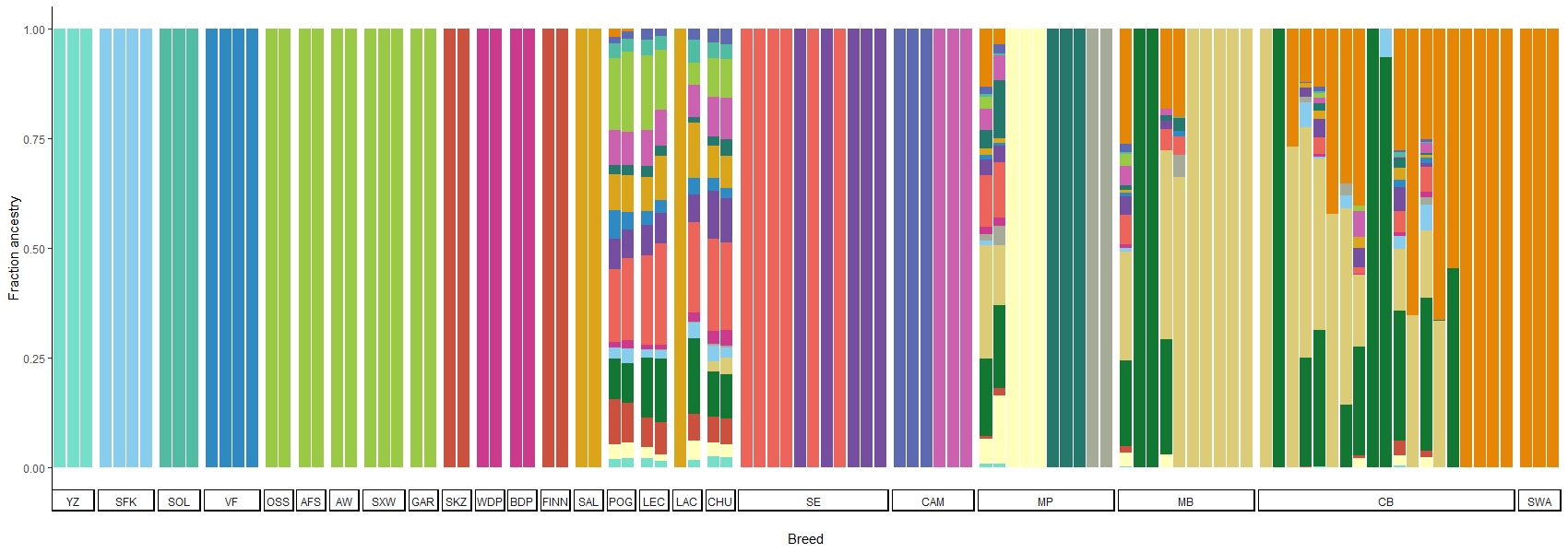

Supplement: Supplementary file 6 [file DataSheet1.ZIP › k18.jpeg]

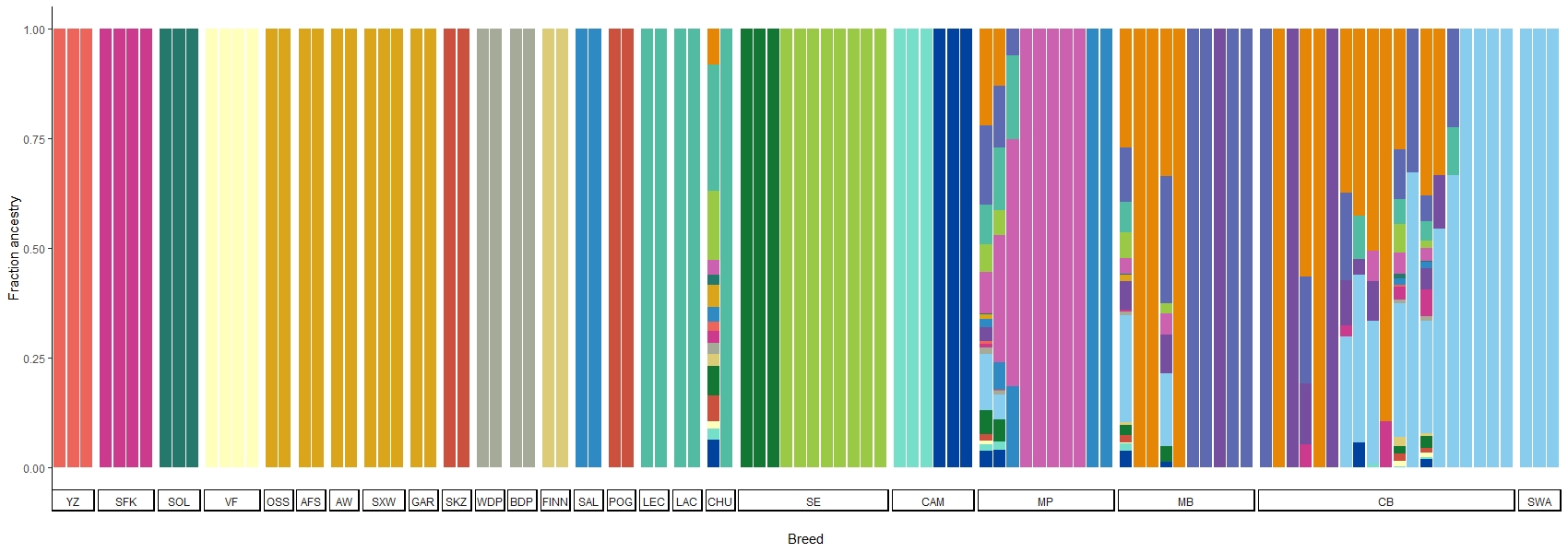

Supplement: Supplementary file 6 [file DataSheet1.ZIP › k19.jpeg]

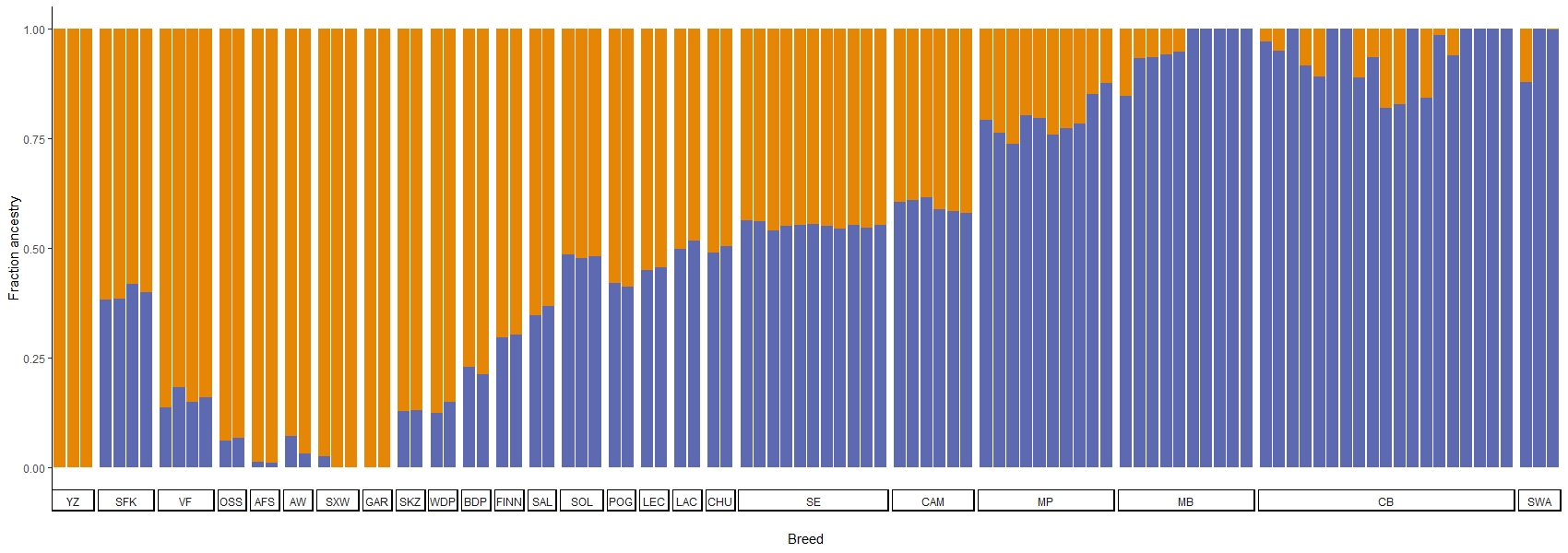

Supplement: Supplementary file 6 [file DataSheet1.ZIP › k2.jpeg]

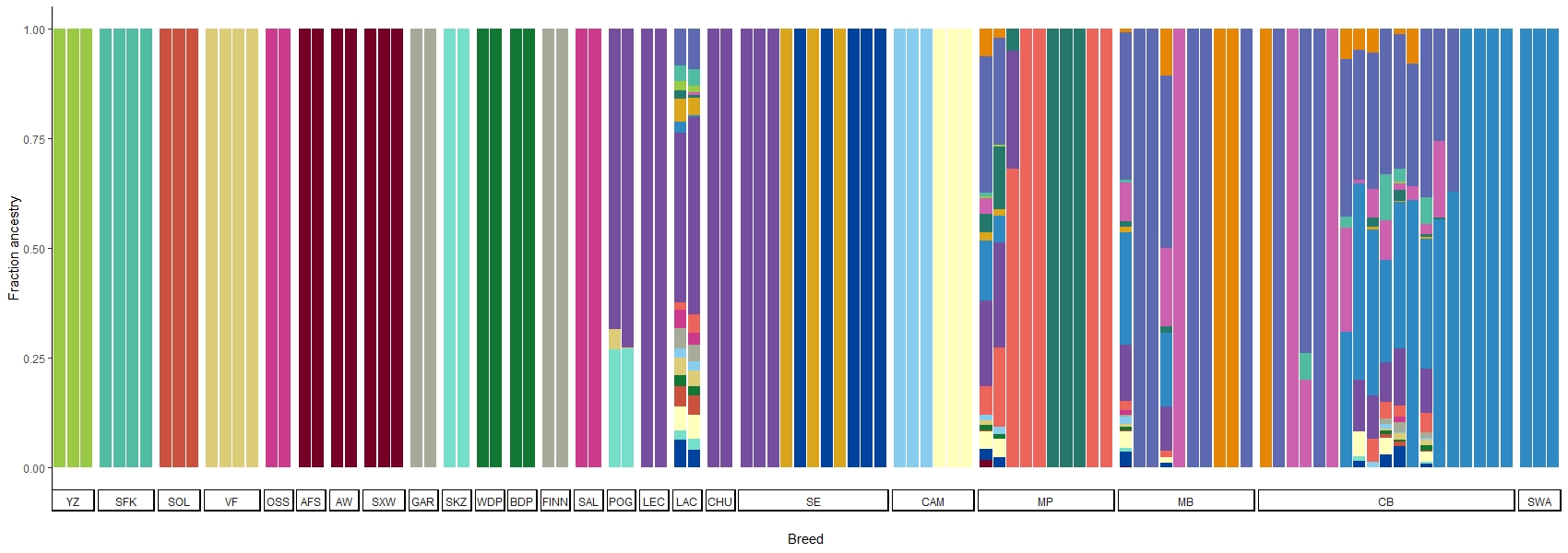

Supplement: Supplementary file 6 [file DataSheet1.ZIP › k20.jpeg]

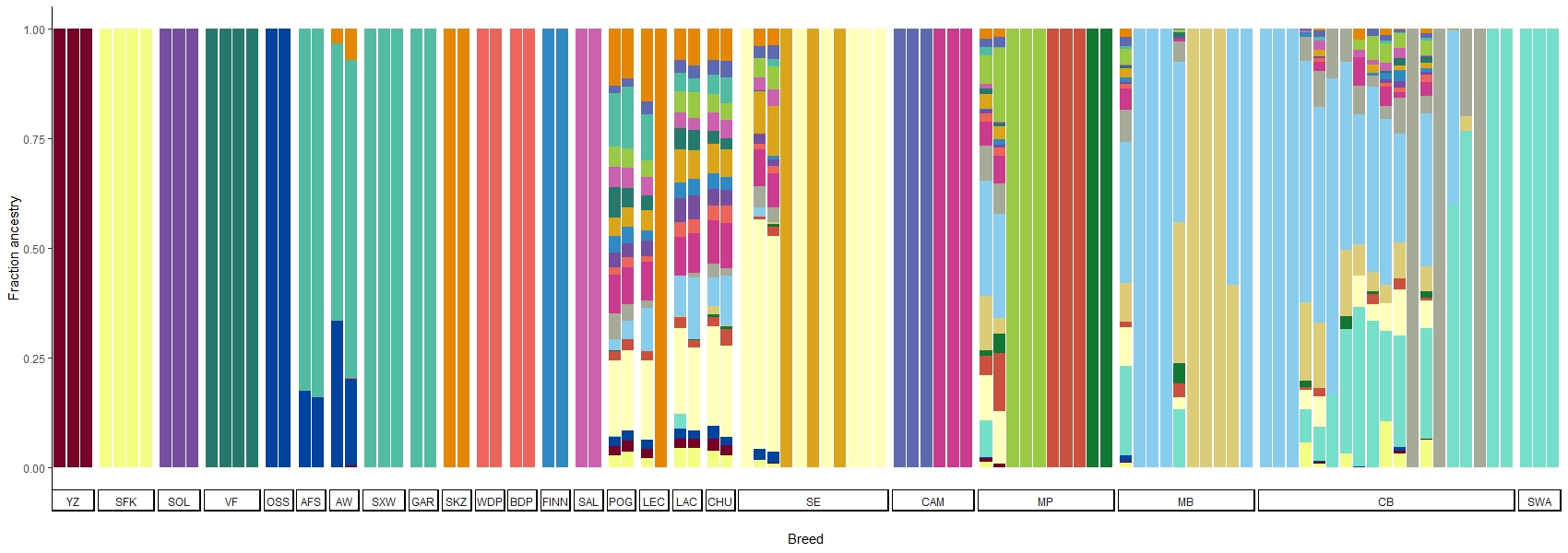

Supplement: Supplementary file 6 [file DataSheet1.ZIP › k21.jpeg]

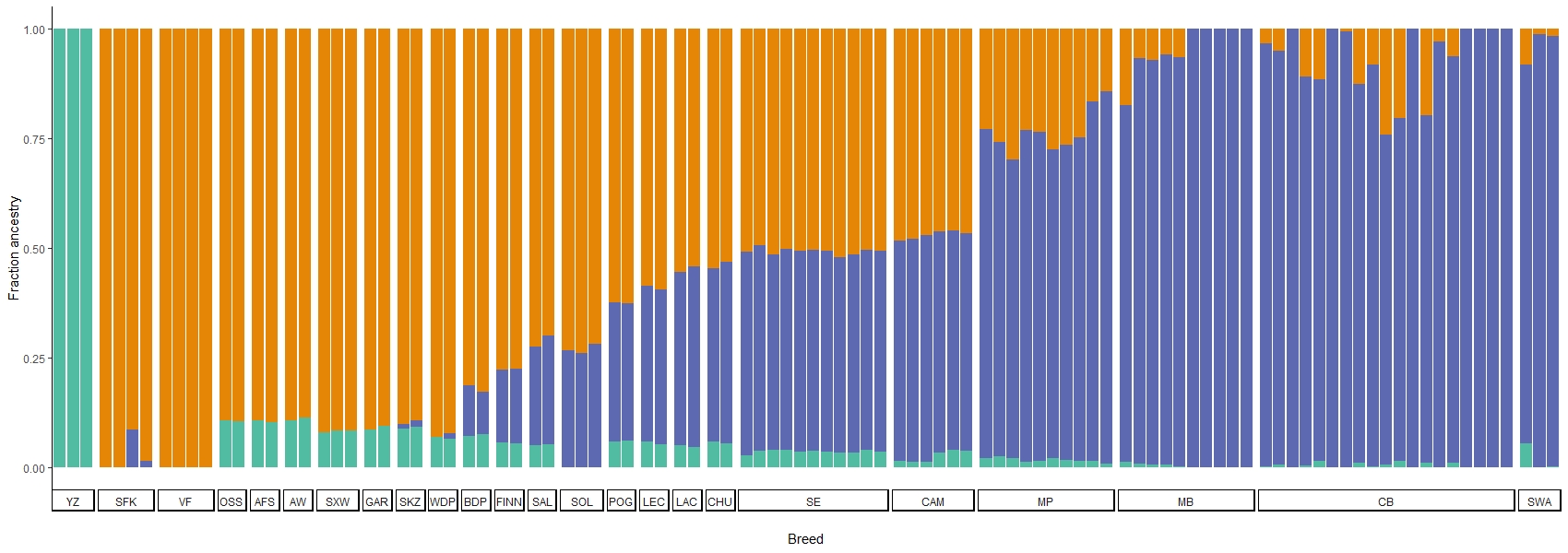

Supplement: Supplementary file 6 [file DataSheet1.ZIP › k3.jpeg]

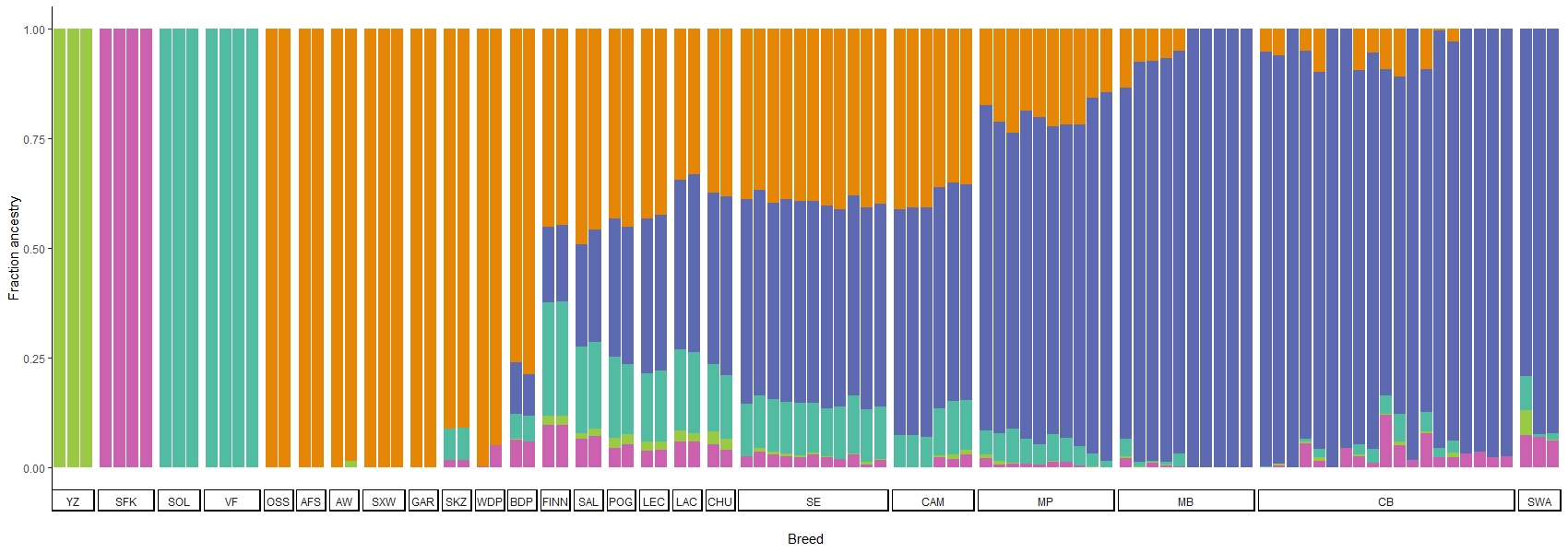

Supplement: Supplementary file 6 [file DataSheet1.ZIP › k5.jpeg]

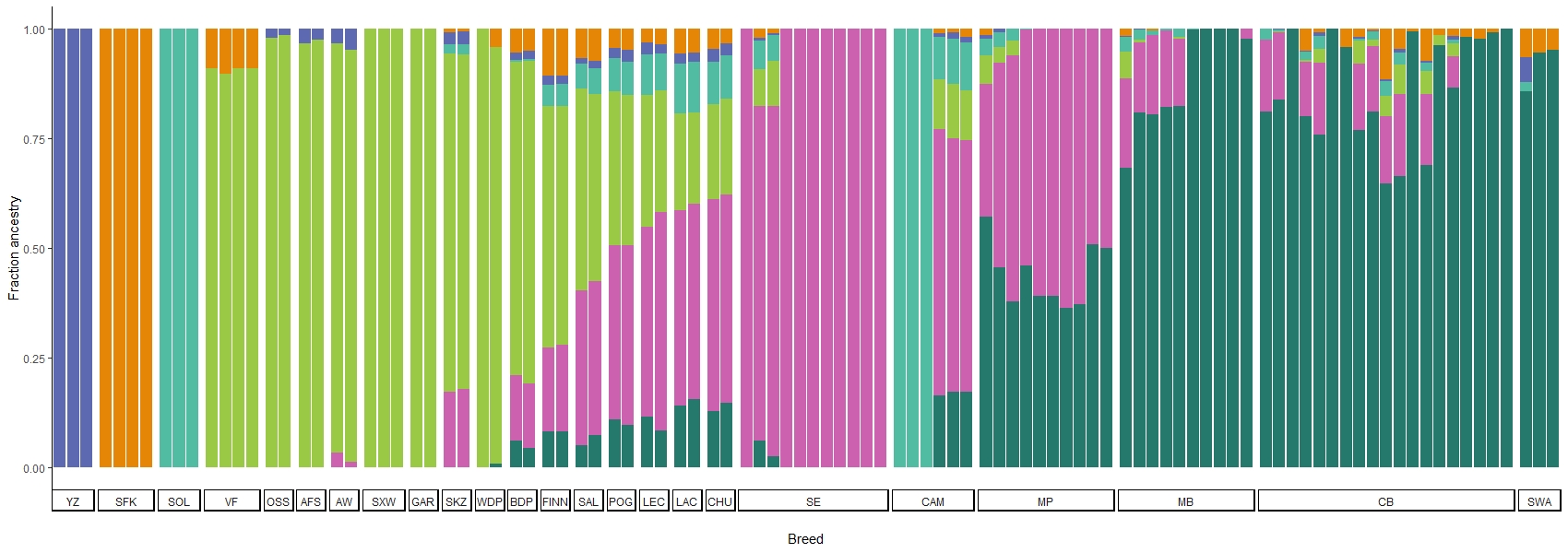

Supplement: Supplementary file 6 [file DataSheet1.ZIP › k6.jpeg]

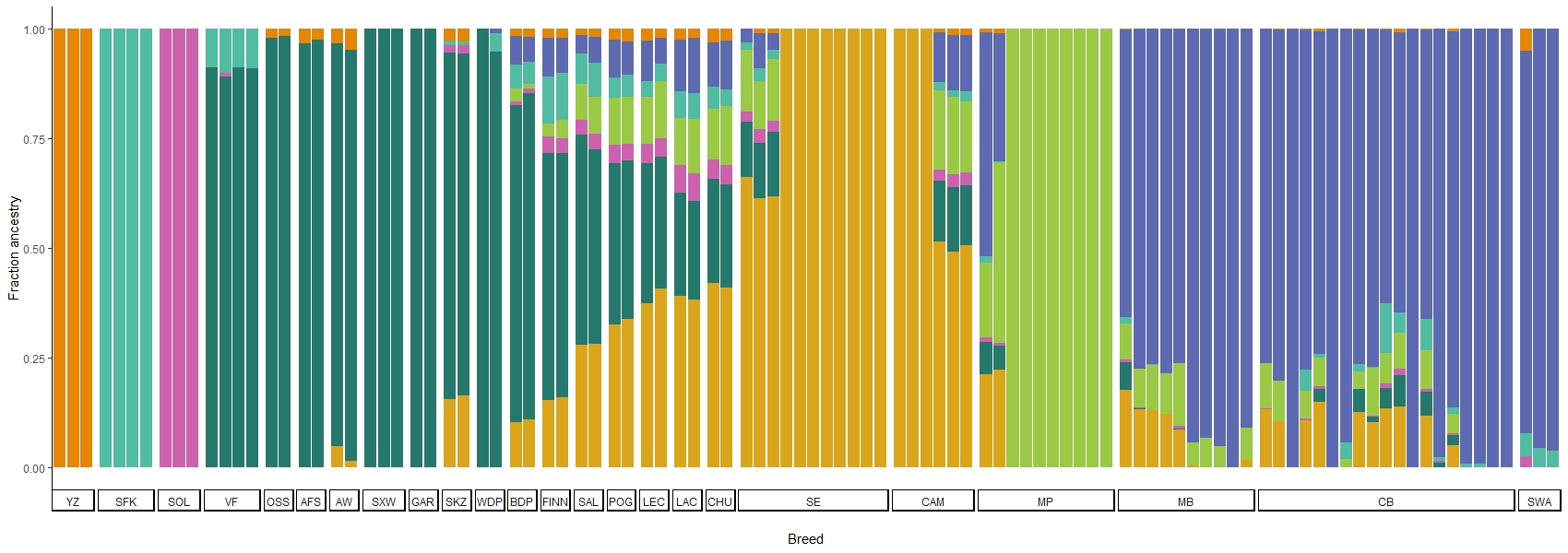

Supplement: Supplementary file 6 [file DataSheet1.ZIP › k7.jpeg]

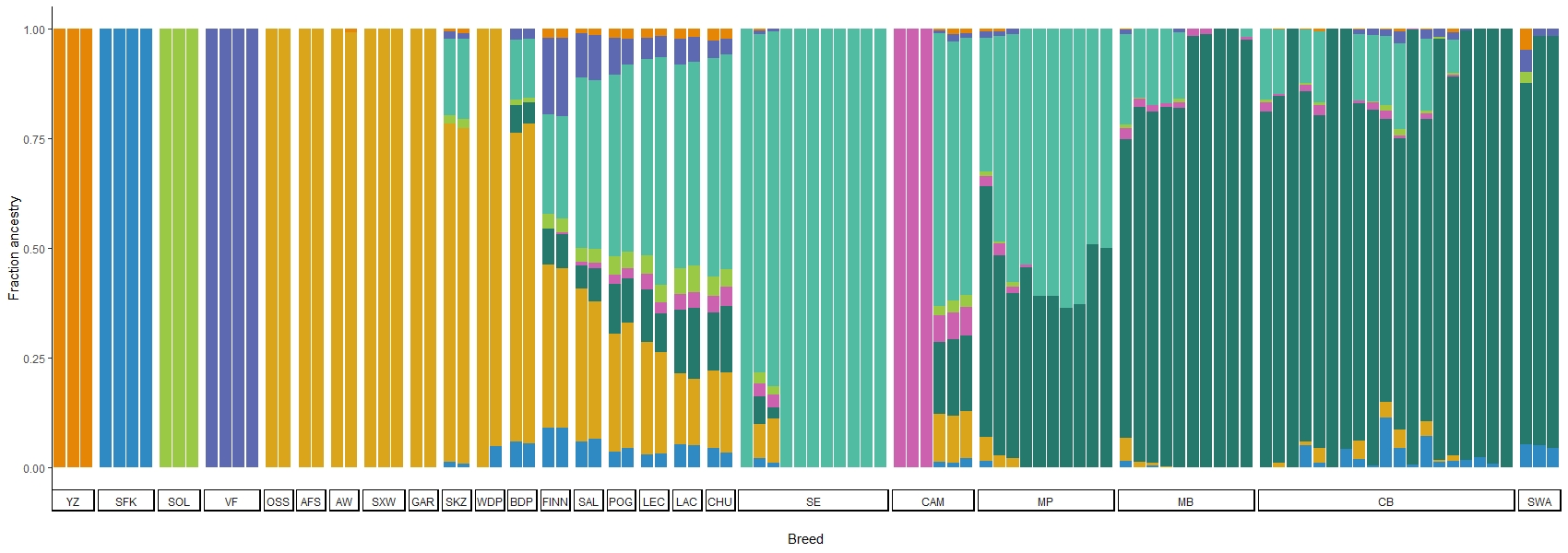

Supplement: Supplementary file 6 [file DataSheet1.ZIP › k8.jpeg]

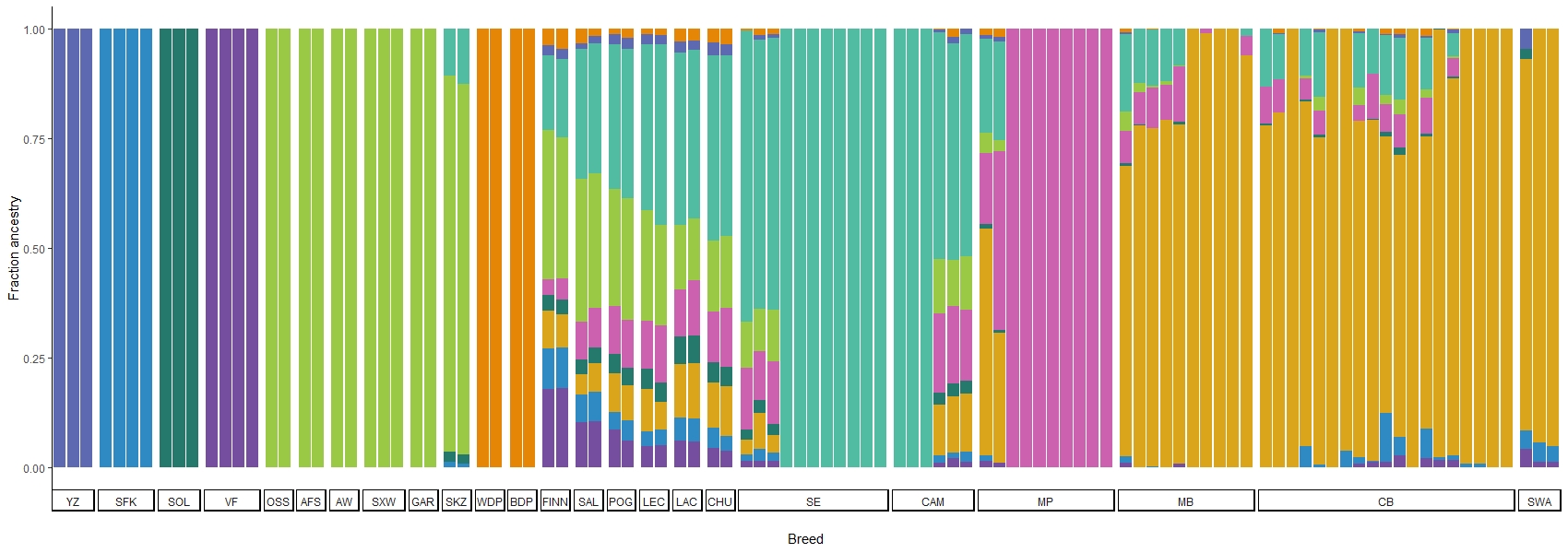

Supplement: Supplementary file 6 [file DataSheet1.ZIP › k9.jpeg]

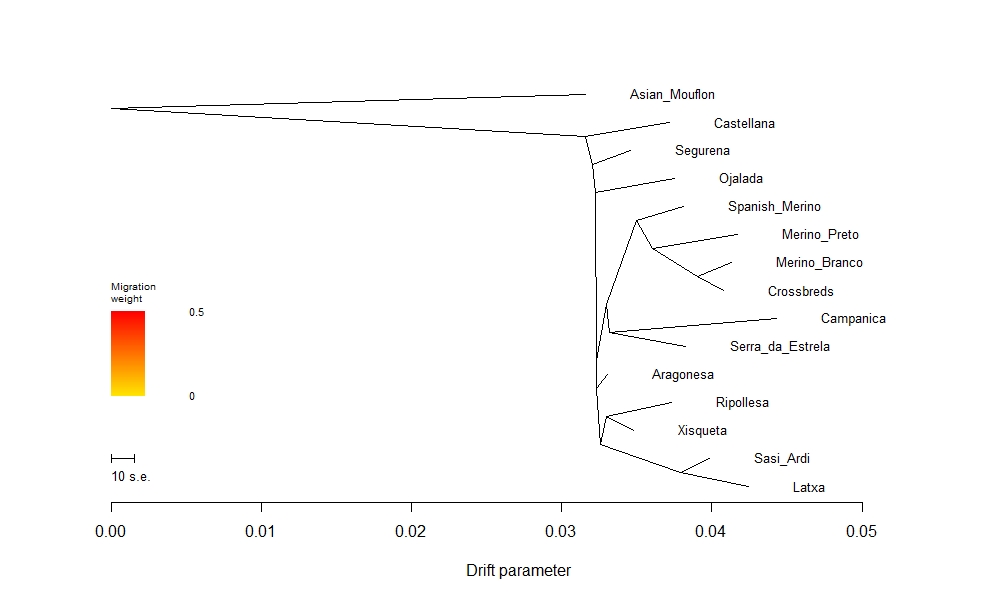

Supplement: Supplementary file 7 [file Image2.JPEG]

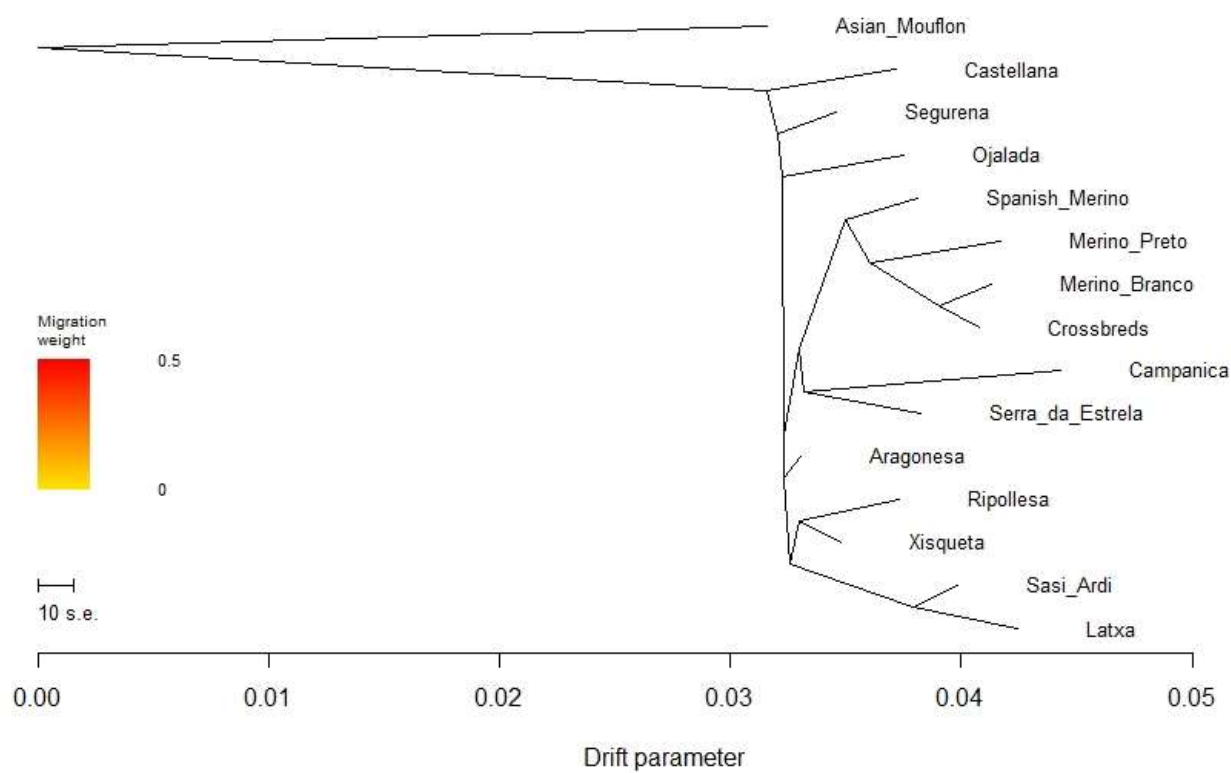

**Figure S4:** Phylogenetic network inferred by Treemix for Iberian sheep for  $m=0$ .

Supplement: Supplementary file 8 [file Image4.pdf]

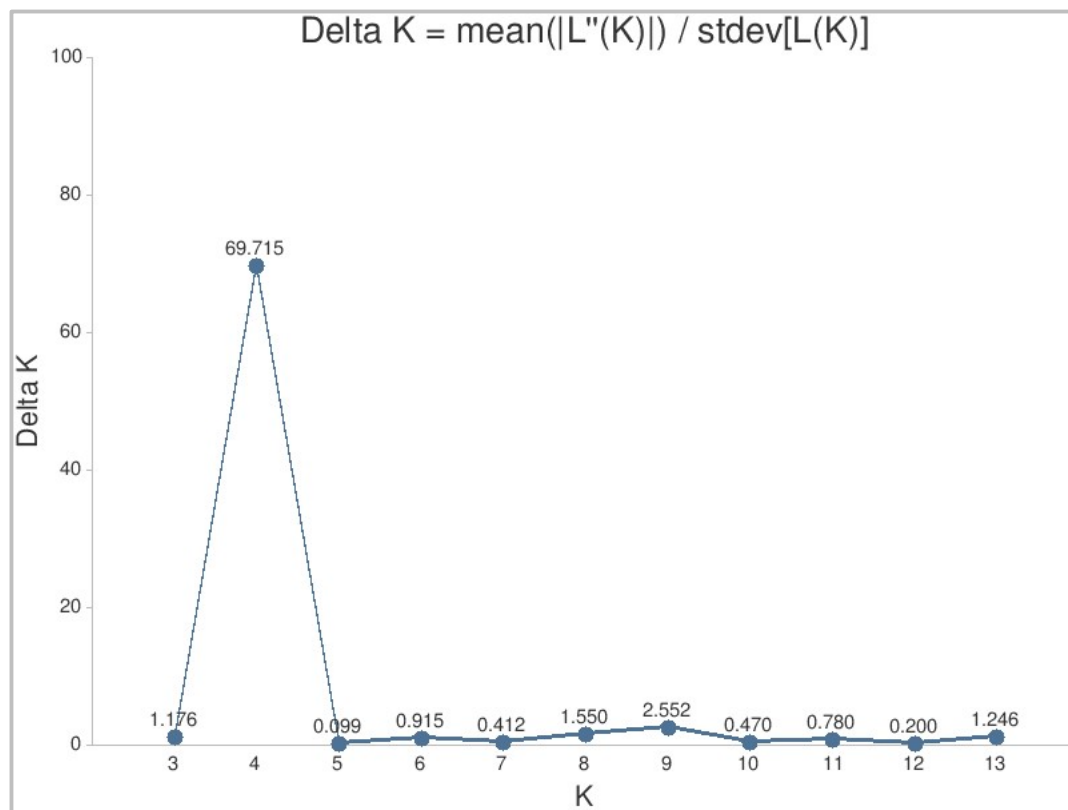

**Figure S2:** Delta K graph obtained with the CLUMPAK software

Supplement: Supplementary file 9 [file Image2.pdf]
