# Supplementary material for: Genome-wide assessment of the population structure and genetic diversity of four Portuguese native sheep breeds
Source: Front Genet. 2023 Jan 13;14:1109490. doi: 10.3389/fgene.2023.1109490 (PMC9880275; doi:10.3389/fgene.2023.1109490)
Supplement: Supplementary file 10 [file Image3.pdf]

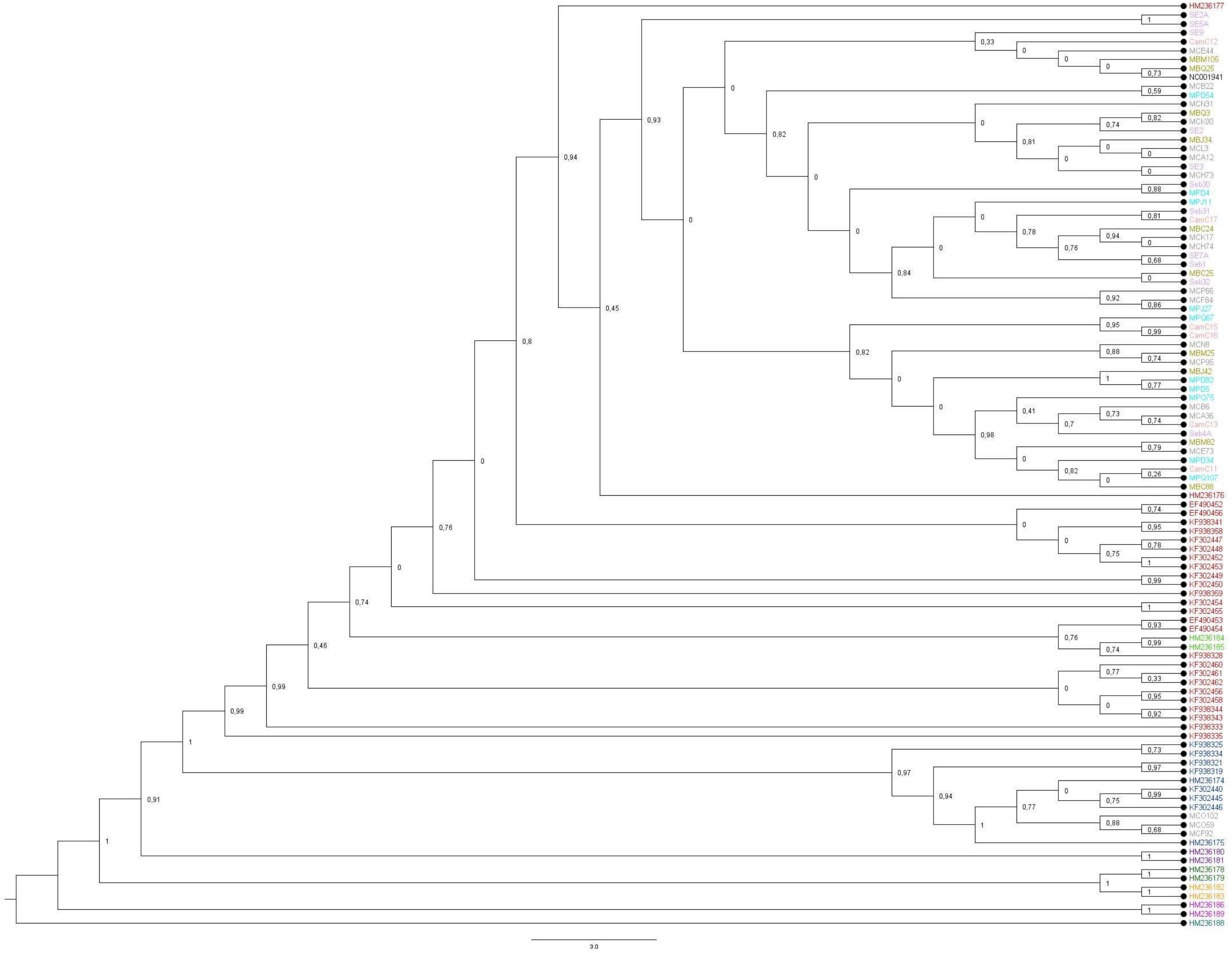

Figure S3: Maximum-Likelihood phylogeny of sheep mitogenomes including accession numbers for all samples.
