# Supplementary material for: Genome-wide assessment of the population structure and genetic diversity of four Portuguese native sheep breeds
Source: Front Genet. 2023 Jan 13;14:1109490. doi: 10.3389/fgene.2023.1109490 (PMC9880275; doi:10.3389/fgene.2023.1109490)
Supplement: Supplementary file 11 [file Table3.pdf]

Table S3: Iberian sheep Illumina Ovine 50 K SNP genotype data included in the analysis.

Breed name	Sample size	Country	Region	Wool Type	Main Purpose	Reference
Castellana	21	Spain	Castile and Leon	Intermediate	meat	
Latxa	24	Spain	Basque Country	Coarse wool	dairy	
Merino	13	Spain	Extremadura	Merino	meat	
Ojalada	23	Spain	Castile and Leon	Intermediate	meat	
Rasa Aragonesa	20	Spain	Aragon	Intermediate	meat	(28)
Ripollesa	21	Spain	Catalonia	Intermediate	meat	
Sasi Ardi	24	Spain	Basque Country	Coarse wool	meat	
Segurena	12	Spain	Andalusia	Intermediate	meat	
Xisqueta	24	Spain	Catalonia	Intermediate	meat	
