# Supplementary material for: Genome-wide assessment of the population structure and genetic diversity of four Portuguese native sheep breeds
Source: Front Genet. 2023 Jan 13;14:1109490. doi: 10.3389/fgene.2023.1109490 (PMC9880275; doi:10.3389/fgene.2023.1109490)
Supplement: Supplementary file 12 [file DataSheet1.pdf]

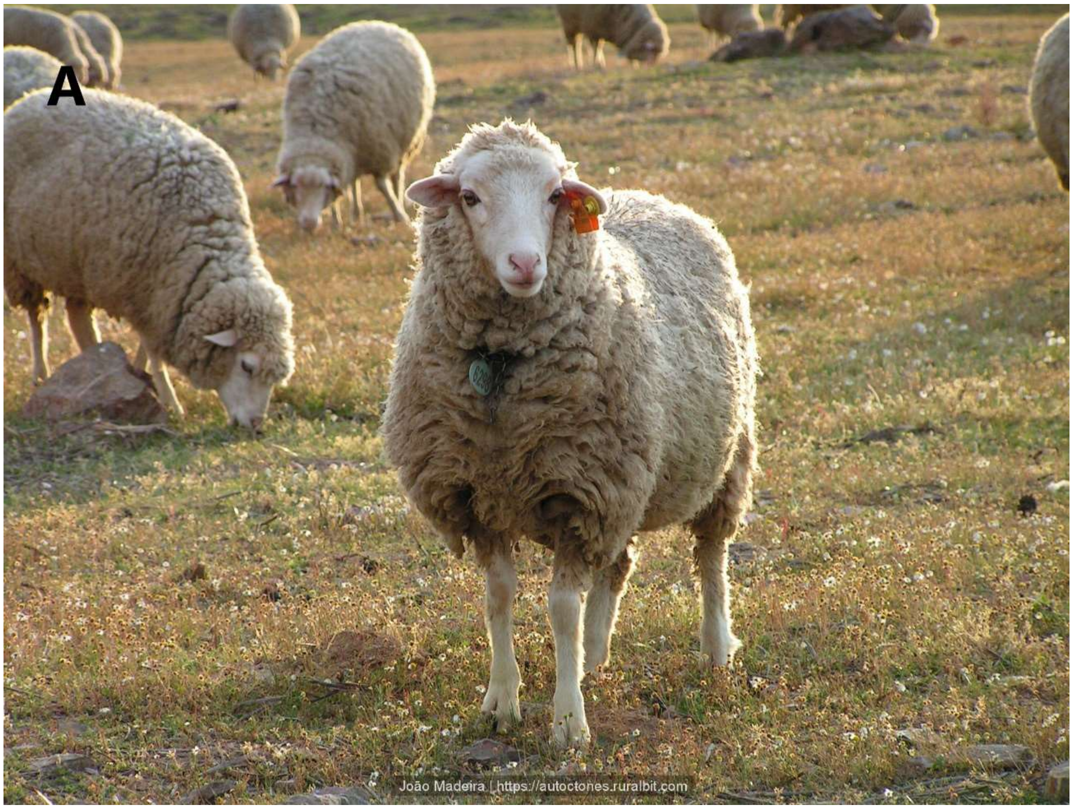
A
João Madeira | https://autoctones.ruralbit.com

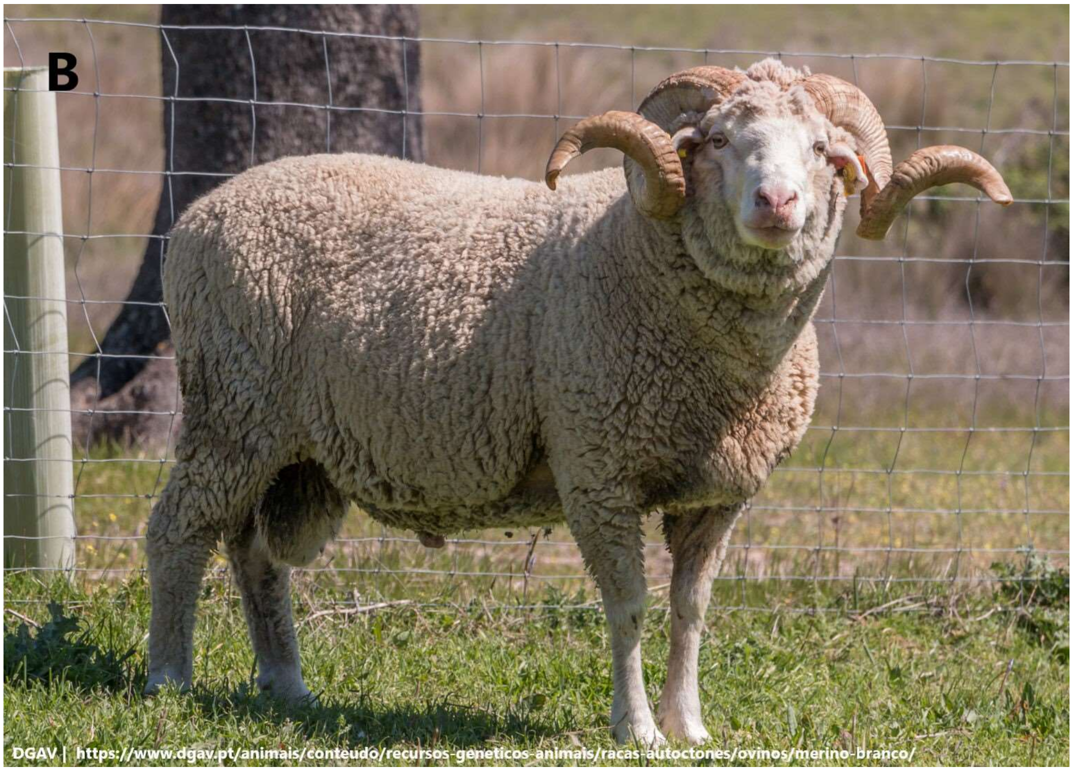
B
DGAV | https://www.dgav.pt/animais/conteudo/recursos-geneticos-animais/racas-autoctones/ovinos/merino-branco/

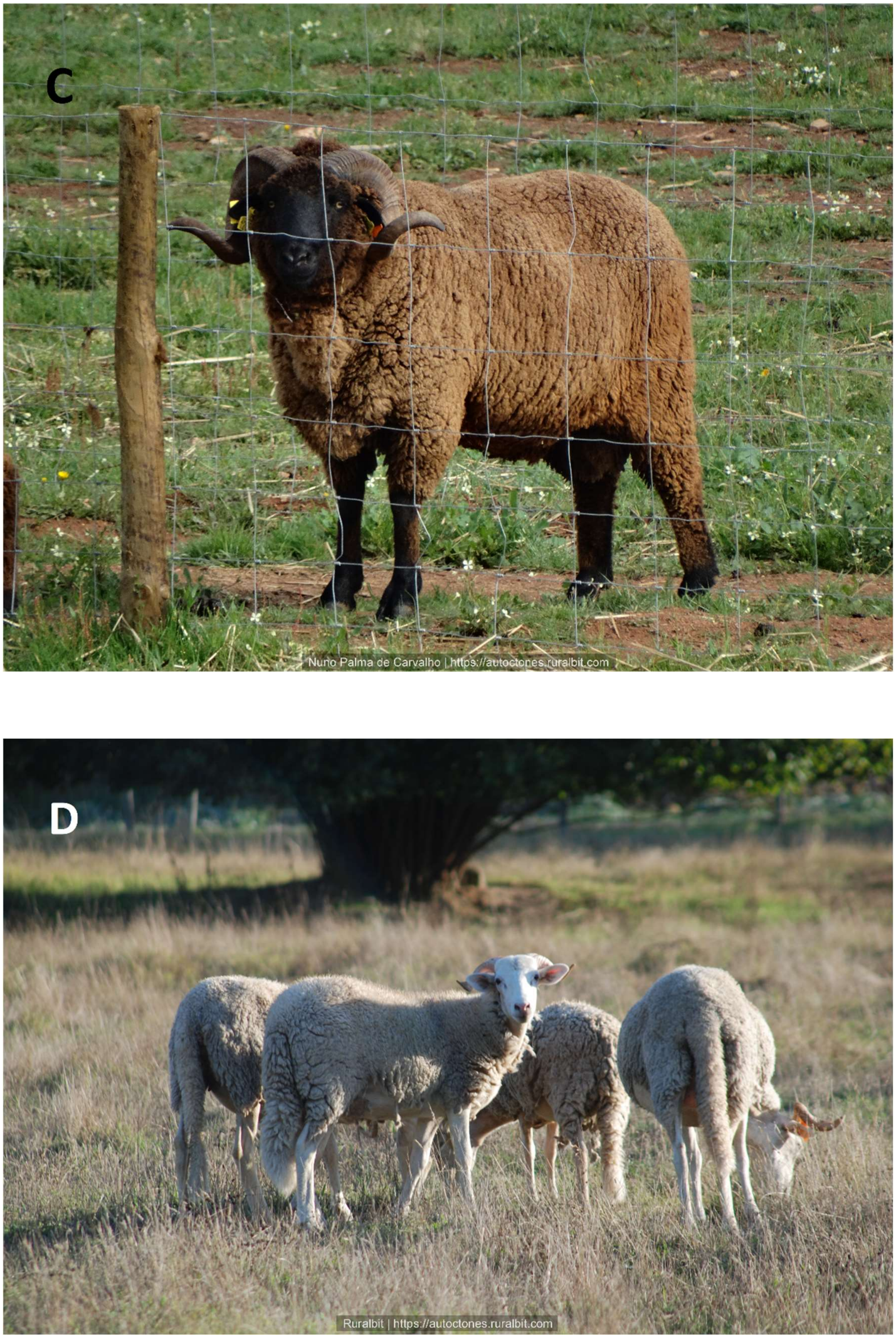

Supplementary File S1: Pictures of the Portuguese native breeds included in the analysis. A – Campaniça; B) Merino Branco; C) Merino Preto; D) Bordaleira Serra da Estrela
