# Supplementary material for: Genome-wide assessment of the population structure and genetic diversity of four Portuguese native sheep breeds
Source: Front Genet. 2023 Jan 13;14:1109490. doi: 10.3389/fgene.2023.1109490 (PMC9880275; doi:10.3389/fgene.2023.1109490)
Supplement: Supplementary file 13 [file Image1.pdf]

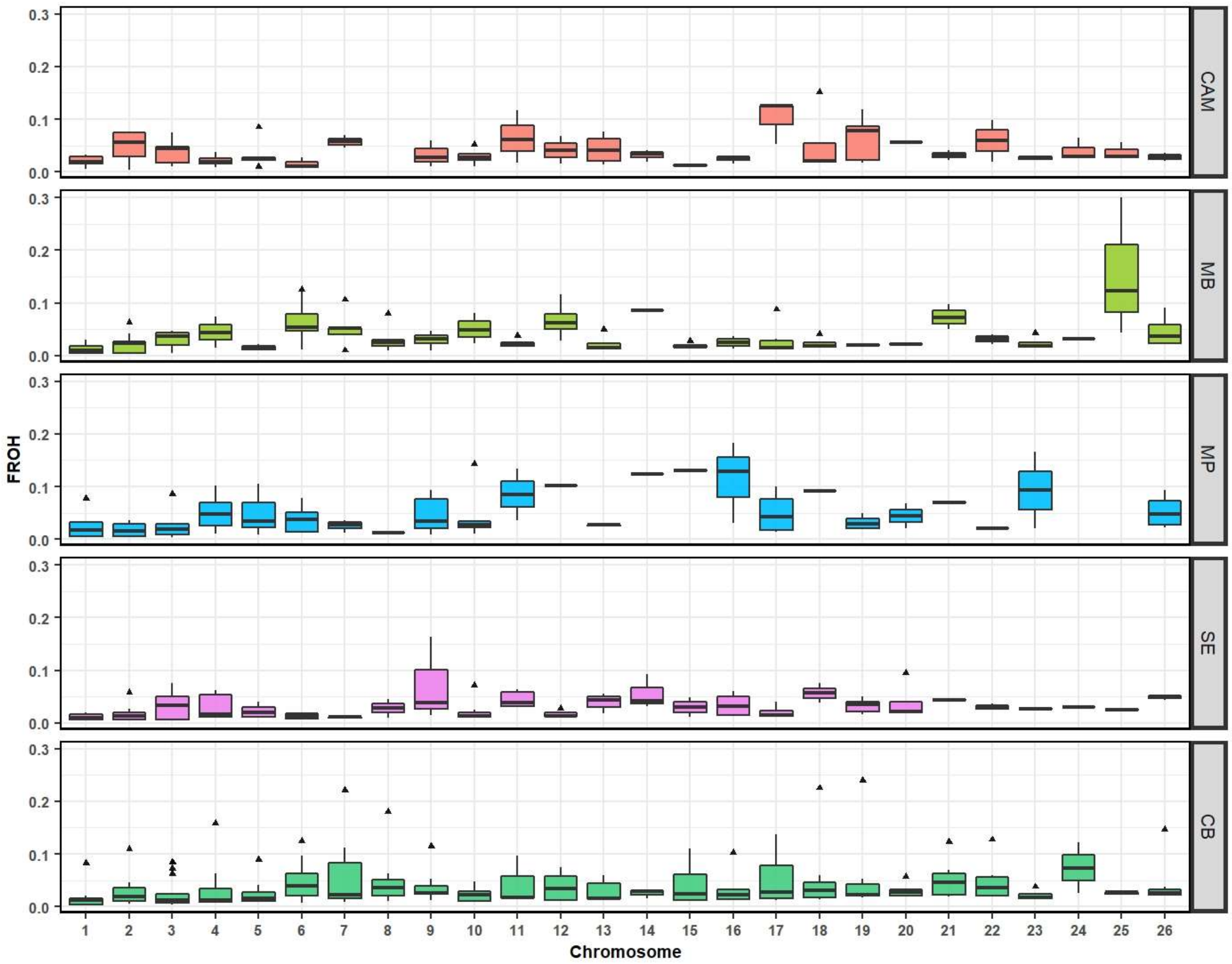

Figure S1: Boxplots depicting the distribution of ROH-based inbreeding coefficient (FROH) per chromosome in each population considering all ROH segments (> 1Mb). CAM – Campaniça; MB – Merino branco; MP – Merino Preto; MC – Crossbreds; SE – Serra da Estrela
